# Interactive Effects of Mycorrhizae, Soil Phosphorus, and Light on Growth and Induction and Priming of Defense in *Plantago lanceolata*

**DOI:** 10.3389/fpls.2021.647372

**Published:** 2021-03-23

**Authors:** Laiye Qu, Minggang Wang, Arjen Biere

**Affiliations:** ^1^State Key Laboratory of Urban and Regional Ecology, Research Center for Eco-Environmental Science, Chinese Academy of Sciences, Beijing, China; ^2^College of Resources and Environment, University of Chinese Academy of Sciences, Beijing, China; ^3^College of Forestry, Beijing Forestry University, Beijing, China; ^4^Department of Terrestrial Ecology, Netherlands Institute of Ecology (NIOO-KNAW), Wageningen, Netherlands

**Keywords:** defense priming, *Funneliformis mosseae*, induced systemic resistance, iridoid glycosides, *Mamestra brassicae*, mycorrhiza-induced resistance, shading, soil phosphorus

## Abstract

Increasing demands to reduce fertilizer and pesticide input in agriculture has triggered interest in arbuscular mycorrhizal fungi (AMF) that can enhance plant growth and confer mycorrhiza-induced resistance (MIR). MIR can be based on a variety of mechanisms, including induction of defense compounds, and sensitization of the plant’s immune system (priming) for enhanced defense against later arriving pests or pathogens signaled through jasmonic acid (JA). However, growth and resistance benefits of AMF highly depend on environmental conditions. Low soil P and non-limiting light conditions are expected to enhance MIR, as these conditions favor AMF colonization and because of observed positive cross-talk between the plant’s phosphate starvation response (PSR) and JA-dependent immunity. We therefore tested growth and resistance benefits of the AMF *Funneliformis mosseae* in *Plantago lanceolata* plants grown under different levels of soil P and light intensity. Resistance benefits were assessed in bioassays with the leaf chewing herbivore *Mamestra brassicae*. Half of the plants were induced by jasmonic acid prior to the bioassays to specifically test whether AMF primed plants for JA-signaled defense under different abiotic conditions. AMF reduced biomass production but contrary to prediction, this reduction was not strongest under conditions considered least optimal for carbon-for-nutrient trade (low light, high soil P). JA application induced resistance to *M. brassicae*, but its extent was independent of soil P and light conditions. Strikingly, in younger plants, JA-induced resistance was annulled by AMF under high resource conditions (high soil P, ample light), indicating that AMF did not prime but repressed JA-induced defense responses. In older plants, low soil P and light enhanced susceptibility to *M. brassicae* due to enhanced leaf nitrogen levels and reduced leaf levels of the defense metabolite catalpol. By contrast, in younger plants, low soil P enhanced resistance. Our results highlight that defense priming by AMF is not ubiquitous and calls for studies revealing the causes of the increasingly observed repression of JA-mediated defense by AMF. Our study further shows that in our system abiotic factors are significant modulators of defense responses, but more strongly so by directly modulating leaf quality than by modulating the effects of beneficial microbes on resistance.

## Introduction

The use of beneficial microbes such as arbuscular mycorrhizal fungi (AMF) is considered a promising way to meet the increasing demand to reduce fertilizer and pesticide use in agriculture and horticulture (Choudhary et al., 2016). AMF form symbioses with over 80% of the land plants, trading mineral nutrients, and especially phosphorus, for carbon sources with their host plants (Smith and Read, 2008). Under a range of conditions, AMF can provide benefits to plants in the form of e.g., growth promotion, enhancement of abiotic and biotic stress tolerance, enhanced pollinator service, and enhancement of tolerance and defense against particular groups of pathogens and herbivores (Bennett et al., 2006; Pozo and Azcon-Aguilar, 2007; Gehring and Bennett, 2009; Hartley and Gange, 2009; Koricheva et al., 2009; Vannette and Hunter, 2009; Jung et al., 2012; Biere and Bennett, 2013; Tao et al., 2016).

The systemic enhancement of resistance against above- and belowground pathogens and insect herbiores by AMF is referred to as “mycorrhiza-induced resistance” (MIR). Several mechanisms underlying MIR have been identified (Cameron et al., 2013). Most prominently, AMF have been shown to sensitize the immune system of their host plants, resulting in a primed state (Conrath et al., 2015; Mauch-Mani et al., 2017). This allows mycorrhizal plants to respond faster and/or stronger when they are challenged by later arriving herbivores and pathogens that are signaled by the plant through the plant hormones jasmonic acid (JA) and ethylene (ET; Pozo and Azcon-Aguilar, 2007; Jung et al., 2012; Song et al., 2013), notably necrotrophic pathogens and generalist chewing leaf herbivores. Priming prepares plants for future attack without directly activating costly defenses (Martinez-Medina et al., 2016). JA-dependent priming forms the basis of induced systemic resistance (ISR) triggered by a variety of beneficial soil microbes (Van Wees et al., 2008; Zamioudis and Pieterse, 2012; Pieterse et al., 2014; Miozzi et al., 2019; Yu et al., 2019; Nishad et al., 2020). In addition to priming of JA-signaled defenses, MIR can also be simply mediated by induction of different classes of defense metabolites that has been widely observed in response to AMF colonization (e.g., Andrade et al., 2013; Vannette et al., 2013; Shrivastava et al., 2015).

Although mycorrhiza-induced resistance has been shown in many systems, it is not a ubiquitously observed phenomenon. For instance, priming of JA-dependent proteinaceous defenses by AMF has been elegantly demonstrated in cultivated tomato (Song et al., 2013), but mixed results have been obtained for wild solanaceous species (Minton et al., 2016). Moreover, even when AMF trigger defense responses, this does not necessarily result in enhanced resistance. This is because AMF colonization commonly results in a diverse set of phenotypic changes in their host plants, that each can contribute to either enhanced resistance or to enhanced susceptibility to a particular pest or pathogen. For instance, AMF colonization results in a strong reprogramming of the root and shoot metabolome (Kaur and Suseela, 2020), that often not only encompassess the induction of defense metabolites, but also results in an enhancement of the nutritional status of root and shoot tissues. Likewise, the set of distinct morphological and physiological changes that are induced by AMF colonization includes alterations in phenology, size, apparency, availability, palatability, and digestibility of plant tissues, that each can either enhance or reduce the plant’s resistance to pathogens and pests (Bennett et al., 2006).

Moreover, both the extent of AMF-induced growth promotion and the extent of MIR have been shown to strongly depend on the abiotic environmental context. This context dependency has been identified as one of the major problems in the application of beneficial microbes to enhance crop resistance and production (Barber et al., 2013). Much of the current research is therefore focused on understanding such context dependency (Koricheva et al., 2009; Hoeksema et al., 2010; Johnson et al., 2015; Pozo et al., 2015). Two of the environmental factors that have been identified as important drivers of context-dependency in both the mycorrhizal growth response (MGR, the percent growth benefit incurred by AMF compared to non-mycorrhizal, NM, plants) and in MIR are the availability of soil phosphorus (Johnson et al., 2015) and light (Konvalinkova and Jansa, 2016).

Optimal trade principles predict that plant growth will most strongly benefit from symbiosis with AMF under conditions of low soil phosphorus supply, when plants gain optimal benefit from the P provided by the fungal partner, and under conditions of ample light, entailing minimal costs for the plant to provide carbon sources in the form of hexoses and fatty acids in return to the fungus (Hoeksema et al., 2010; Johnson, 2010). Indeed, in an elegant series of grassland experiments, Johnson et al. (2015) showed that mutualisms between *Andropogon gerardii* and AMF arose in P-limited but not in N-limited systems, and that shading generated less mutualistic interactions, resulting in reduced plant and fungal biomass. These results are corroborated by several experimental studies showing that the growth benefit of AMF is strongly reduced, or even becomes negative, under increasing levels of soil P (Hetrick et al., 1983), as well as under conditions of shading (reviewed by Konvalinkova and Jansa, 2016). Accordingly, plants have evolved active mechanisms to reduce colonization by AMF under such unfavorable conditions for trade. The AM symbiosis is tightly regulated and integrated with the plant’s Pi status through a complex signaling network, involving phytohormones, micro-RNAs, and secreted peptides (Vierheilig et al., 2000; Nagy et al., 2009; Mueller and Harrison, 2019) in a process known as autoregulation of mycorrhization (AOM). In this process, Pi acts systemically to repress the expression of symbiotic genes, such as genes involved in the production of strigolactones, important in the initial plant-AMF dialog, and symbiosis-associated phosphate transporters (Breuillin et al., 2010), resulting in lower AMF colonization and functionality at high soil phosphate and high internal plant Pi levels (Nagy et al., 2009; Smith and Smith, 2011; Gosling et al., 2013). Similarly, low light levels (shading) commonly result in lower levels of AMF colonization (reviewed by Konvalinkova and Jansa, 2016).

Not only plant growth, but also MIR is expected to be affected by soil P and light availability, for two reasons. First, as explained above, plants will tend to reduce colonization rates by AMF under conditions of high soil P and shading, which may reduce the extent to which MIR can be activated. Second, recent studies show that soil P can be an important driver of plant immunity. Phosphate deficiency activates the phosphate starvation response (PSR) in plants (Morcuende et al., 2007; Bustos et al., 2010; Rouached et al., 2010; Chiou and Lin, 2011). Recent studies have shown strong cross-talk between the signaling pathways involved in PSR and immunity. High expression of the master transcriptional regulators of PSR in *Arabidopsis thaliana*, *PSR 1 (PHR1)*, and *PHR1-LIKE 1 (PHL1)* enhances the expression of jasmonic acid (JA)-inducible genes associated with defense against generalist leaf chewing insect herbivores and necrotrophic pathogens, but directly represses salicylic acid (SA)-inducible genes, associated with defense against biotrophic pathogens and sap-sucking or cell-feeding insects (Castrillo et al., 2017). This pattern is confirmed by bioassays with insect herbivores and plant pathogens. Several plant species, including *A. thaliana*, tomato, and tobacco, were shown to induce the JA pathway and enhance defense against a generalist leaf chewing insect herbivore when they experienced deficiency in inorganic phosphate (Pi; Khan et al., 2016), a response that was attenuated in JA- as well as PSR-signaling mutants (Khan et al., 2016). By contrast, functional PSR signaling was shown to be associated with enhanced susceptibility to a bacterial and an oomycete pathogen in *A. thaliana* (Castrillo et al., 2017). This is in agreement with earlier studies showing that several transcription factors co-regulate both PSR and immunity (Baek et al., 2017). For instance, *WRKY75* not only activates transcription of PSR genes (Devaiah et al., 2007), but also increases the transcript levels of marker genes of the JA defense signaling pathway, whereas it decreases the expression of marker genes of the SA defense signaling pathway (Schmiesing et al., 2016), or by AMF-mediated recruitment of ISR-inducing rhizobacteria (Cameron et al., 2013).

Collectively, these studies suggest that low soil phosphate may contribute to enhanced plant resistance to leaf chewing herbivores either through enhanced JA immune signaling or through enhanced functional association with ISR-inducing beneficial microbes. However, only few studies have studied interactions between MIR and soil P to test whether low soil P can indeed enhance MIR. In support of this idea, AMF did not induce resistance to charcoal rot in soybean under high soil P conditions, but incurred a more than 5-fold reduction in disease severity under low soil P, that more than compensated the 2.5-fold increase in susceptibility caused by low P in the absence of AMF (Spagnoletti et al., 2018). By contrast, in susceptible wheat, AMF reduced mildew severity independent of soil P, despite its lower colonization rate at high soil P, and low P itself reduced mildew severity as well (Mustafa et al., 2016). Even fewer studies have considered interactions between soil P and MIR with respect to resistance against insect herbivory. Wang et al. (2020) observed that low soil P resulted in higher AMF colonization and lower aphid infestation, but combinatory effects were not tested. Furthermore, as far as we know, no studies have investigated how the effects of soil P on MIR depend on the availability of carbohydrates, as affected by the availability of light for photosynthesis. Like high soil P, low light conditions are expected to hamper plant colonization by resistance-inducing AMF, and may also limit C-resources for the production of defense metabolites upon activation of defenses. Low light conditions may thus counteract the positive effects of low P on MIR.

In this paper, we study the interactive effects of AMF, soil P, and light intensity on plant growth and MIR against a generalist leaf chewing insect herbivore. We grew plants of ribwort plantain (*Plantago lanceolata*) under a factorial combination of light and soil P conditions in the presence or absence of the AM fungus *Funneliformis mosseae*. Subsequently, we used bioassays with caterpillars of the generalist leaf chewing insect herbivore *Mamestra brassicae* to test effects of light, soil P, and AMF on resistance. To specifically test whether AMF can prime plants for JA-dependent defenses and whether such effects are modulated by soil P and light, we challenged half of the plants with leaf application of JA prior to the bioassays. In addition, we measured a set of leaf biochemical and morphological leaf traits of bioassay plants to enable us to associate changes in resistance with changes in leaf traits.

Of specific interest are a class of carbon-based terpenoid defense metabolites produced by *P. lanceolata*, known as iridoid glycosides (IGs), that commonly deter generalist chewing insect herbivores (Puttick and Bowers, 1988; Harvey et al., 2005; Reudler et al., 2011), but can also reduce the performance of fungal pathogens (Marak et al., 2002a; Biere et al., 2004) and AMF (De Deyn et al., 2009). IGs in *P. lanceolata* can be induced by a wide range of organisms, including not only generalist and specialist insect herbivores (e.g., Bowers and Stamp, 1993; Darrow and Bowers, 1999; Fuchs and Bowers, 2004; Quintero and Bowers, 2011, 2012), but also pathogens (Marak et al., 2002b) and AMF (Gange and West, 1994; Bennett et al., 2009; Schweiger et al., 2014a; Wang et al., 2015). Based on stoichiometric principles, production of C-based secondary metabolites such as IGs is expected to be reduced under low light, high nutrient conditions (Fajer et al., 1992) and this is supported for IGs in *P. lanceolata* by a number of studies (Fajer et al., 1992; Darrow and Bowers, 1999; Jarzomski et al., 2000; Tamura, 2001; Marak et al., 2003; Miehe-Steier et al., 2015; Pankoke et al., 2015). This could be an additional reason for lower induction or priming of these defenses in response to AMF under low light and high soil P conditions. We examine whether induction or priming of these compounds by AMF is involved in MIR and whether the extent of induction or priming is dependent on soil P and light conditions. Furthermore, given the strong changes in plant defense strategies and responsiveness to induction during ontogeny (Van Dam et al., 2001; Boege and Marquis, 2005; Barton and Koricheva, 2010; Miller et al., 2014), we study whether these effects change with plant age.

We address the following specific questions: (1) Are root colonization rates and effects of the AM fungus *F. mosseae* on growth of *P. lanceolata* affected by light and soil P conditions? (2) Are effects of AMF on induction and priming of JA-signaled plant defenses in *P. lanceolata* against the generalist insect herbivore *M. brassicae* affected by light and soil P? (3) Which biochemical and morphological leaf traits changes are associated with the effects of AMF, light, and P on resistance? We hypothesize that the beneficial effect of AMF on plant growth is highest under conditions optimal for trade (high light, low soil P) and lowest under shading and high soil P, and that the extent of induction or priming of defense is similarly strongest under low soil P and high light conditions.

## Materials and Methods

### Plant Materials and Growing Conditions

Ribwort plantain (*Plantago lanceolata* L.) is a rosette-forming, self-incompatible, wind pollinated, perennial herb. It has a worldwide distribution and is commonly used in studies of plant-AM fungus interactions and plant defense (e.g., Gange and West, 1994; Bennett et al., 2009; De Deyn et al., 2009; Pankoke et al., 2015; Wang et al., 2015). The major defense metabolites produced by *P. lanceolata* are the IGs aucubin and catalpol. Like glucosinolates, IGs form a dual defense system, in which vacuole-stored IGs are activated by membrane-bound beta-glucosidases upon damage (Pankoke et al., 2013). Seeds of *P. lanceolata* were obtained from Cruydthoeck (Assen, Netherlands), a supplier of seeds collected from wild plant populations. Seeds were surface-sterilized in a 1% sodium hypochlorite solution for 15 min, rinsed with demineralized water, and germinated on moistened glass beads in a growth cabinet at 20°C and a 16 h photoperiod for 12 days. Seedlings were individually transplanted into 10 cm× 10 cm× 11 cm plastic pots filled with 675 g of a 1:1 (v:v) mixture of sterilized sand (particle size 0.75–1.5 mm) and Sorbix (an inert absorbent, Imerys Industrial Minerals, Fur, Denmark). Pots were placed in 15 × 15 × 7 containers to allow re-absorption of drained water by plants and to prevent contamination of soil biota contained within the drainage water between pots. Plants were grown in a climate-controlled greenhouse at Netherlands Institute of Ecology at 22 ± 2°C and a 16 h photoperiod. Natural daylight was automatically supplemented with light from 400-W metal halide lamps when natural light levels dropped below 225 μmol m^−1^s^−1^ photon flux density.

### AMF, Soil P, and Light Treatments

A factorial combination of two AMF treatments (M), two soil phosphorus treatments (P), and two light treatments (L) was applied. As source of AM fungal inoculum, we used *F. mosseae* BEG 198 (Symbiom Ltd., Lanskroun, Czech Republic). This species forms symbioses with many plant species including *P. lanceolata* (Karasawa et al., 2012; Orlowska et al., 2012; Wang et al., 2015). Fifteen grams of inoculum (granules containing hyphae, spores, and substrate) were mixed into pots assigned to the mycorrhizal inoculation treatment (M+) before seedling transplantation. Pots assigned to the non-mycorrhizal inoculation treatment (M−) received 15 g of sterilized *F. mosseae* inoculum. Because AMF usually perform better in a non-sterile background, 30 ml of a microbial wash was added to all (mycorrhizal and non-mycorrhizal) pots before seedling transplantation. Soil used to obtain a microbial wash originated from a grassland on loamy sand soil in a nature restoration area (Renkum, Netherlands, N 52°00'9'', E 5°45'8'') that contained 1.45 g total N kg^−1^ and 0.25 g P_2_O_5_ kg^−1^ soil (Hannula et al., 2017). The microbial wash was obtained by adding 20 kg of soil to 20 L of demineralized water, stirring overnight, and filtering through successively smaller filters (200, 75, 45, and 20 μm) to remove any resident mycorrhizal hyphae and spores, but retaining a microbial background population. Soil phosphorus was applied to experimental pots in the form of bonemeal containing 16% of P_2_O_5_ (Ecostyle B.V., Oosterwolde, Netherlands). Before seedling transplantation, 1 g of bonemeal was mixed into pots assigned to the high soil phosphorus treatment (P+), whereas 0.1 g was added to pots of the low soil phosphorus treatment (P-), corresponding to ca. 105 and 10.5 mg of organically bound P per kg soil, respectively. Shading treatments were applied to groups of 16 plants (four plants from each of the four combinations of AMF and soil P treatment) that were placed together in 100 cm × 70 cm flats. Half of the flats were covered with a light shade cloth that reduced light levels by only 15% (high light, L+), as measured with a Licor Quantum Sensor LI-190SA (Licor, Bad Homburg, Germany). The cloth was mounted 50 cm above pot level, covering both the top and the sides of the flats. The other half of the flats was covered with shade cloth that reduced light levels by 50% (low light, L-). In total, 24 flats were used, that were grouped into 12 blocks, each block consisting of one group of L+ plants and one group of L-plants. In total, 480 plants were grown (12 blocks × 2 M × 2 P × 2 L treatments × 5 replicates per block). Positions of blocks within the greenhouse were rearranged weekly to avoid effects of any spatial differences in light conditions within the greenhouse. Plants received 0.5 strength Hoagland nutrient solution (Hoagland and Arnon, 1950) without any phosphate (KH_2_PO_4_) once a week (increasing from 20 ml. in week 2 to 80 ml. in week 10), as well as additional water to maintain soil moisture levels at around 16%. A total of 192 plants were harvested to monitor growth, biomass allocation, and shoot and root morphology and biochemistry across four time points during plant development (“sequential harvests,” 3, 6, 9, and 12 weeks after seedling transplantation). The remaining 288 plants were used to test plant resistance against the generalist leaf chewing herbivore, *M. brassicae*, at two time points during development (“bioassays,” 7 and 10 weeks after seedling transplantation).

### Sequential Harvests

At each of the four sequential harvests, 48 plants (one from each treatment combination from 3 to 6 of the blocks) were harvested. Plants were separated into roots, caudex, leaves, and (at the fourth harvest, when half of the plants had initiated flowering) inflorescences. Roots were thoroughly rinsed to remove all particles of soil substrate. Root and shoot fractions were then oven dried at 60°C for 48 h to measure their dry weights and the root mass fraction (RMF, root dry weight divided by total dry weight). The MGR was calculated as 100 × (M-NM)/NM (Cavagnaro et al., 2003), where M and NM are the dry weights of mycorrhizal and non-mycorrhizal plants, respectively. AMF colonization was quantified at two time points, 6 and 9 weeks after inoculation with *F. mosseae*. A weighed subsample of fresh root material, <1 mm in diameter, was taken, cut in 1.5 cm pieces, cleared in 10% KOH for 20 min. at 90°C, rinsed, stained in a 5% Parker Blue ink–vinegar solution for 15 min. at 90°C, washed, and destained in a 1:1:1 solution of lactic acid, glycerol, and 1% HCL (Vierheilig et al., 1998). AM colonization was recorded according to the magnified intersections method (McGonigle et al., 1990). The incidence of AM structures (hyphae, arbuscules, and vesicles) was scored at a total of 120 intersections to calculate the incidence (%) of each structure over the total number of intersections and the total percentage of root colonization.

### Bioassays

At two time points, 7 and 10 weeks after seedling transplantation, bioassays were performed (“Bio1” and “Bio2,” respectively) to test plant resistance against the generalist leaf chewing herbivore *M. brassicae*. For each of the bioassays, 144 plants were used (18 per treatment combination, maximum two per treatment combination per block).

#### Insect Rearing

The cabbage moth [*M. brassicae* (Noctuidae, Lepidoptera)] is a polyphagous chewing insect herbivore whose caterpillar larvae feed on a broad range of host plant species in the Palearctic (Rojas et al., 2000). Insect eggs were obtained from the Laboratory of Entomology, Wageningen University, Netherlands. Caterpillars were grown on artificial diet (Singh et al., 1983) until they reached L3 and starved for 12 h prior to the bioassays.

#### JA Treatment

In response to generalist leaf chewing herbivores, such as *M. brassicae*, plants generally activate primarily the JA signaling pathway, resulting in the downstream activation of defenses that are effective to these biotic challenges. JA application to plants is often used to mimic the activation of the JA pathway following attack by generalist chewing insect herbivores (Dicke and Hilker, 2003). To test whether mycorrhizae can prime plants for a faster or stronger response to a biotic challenge that triggers the JA signaling pathway, half of the plants (nine per treatment combination) were challenged by application of JA 48 h. prior to initiating the bioassays. A pipette was used to apply 0.5 ml of a 9.5 mM solution of JA dissolved in 0.1% triton to the sixth (Bio1) or ninth (Bio2) youngest fully expanded leaf of plants assigned to the JA treatment. The solution was dispersed across the leaf by gently spreading it over the leaf surface using gloves. The other half of the plants, serving as control, similarly received 0.5 ml of a 0.1% triton solution. Lower performance of bioassay caterpillars on JA-induced plants with AMF than on JA-induced plants without AMF would indicate *priming* of JA-signaled defenses by AMF. Lower performance of bioassay caterpillars on control plants (not treated with JA) with AMF than on control plants without AMF would indicate *induction* of defenses by AMF.

#### Bioassays

After measuring their initial fresh weight (CFW1), one freshly molted (Bio1) or 2nd day (Bio2) L3 caterpillar was put in a clip cage (5 cm diameter) mounted on the 3rd (Bio1) or 6th (Bio2) youngest leaf of each plant, representing a leaf of intermediate age on plants from these time points, respectively. After 24 (Bio1) or 48 h (Bio2), caterpillars were taken off the plants, re-weighed (CFW2), and dried at 60°C for 48 h to measure their dry weights (CDW2) and dry matter content (CDMC = CDW2/CFW2). Fourteen of the 288 caterpillars died or were lost during the experiment and were excluded from analysis. Leaves used for the bioassays were then removed from the plant, and scanned to estimate the amount of remaining leaf area (LA) and leaf area eaten (dLA) using WINFOLIA (Regent Instruments Inc., Quebec, Canada). In cases where caterpillars fed from leaf margins rather than chewing holes, accurate estimation of the leaf area eaten depends on the ability to reconstruct the leaf margin. This was not possible in 14 of the 288 samples and these data were excluded from analysis. The remains of the leaves were then dried at 60°C for 48 h to measure their dry weights (LDW). For each plant, leaf dry weight eaten (dLDW) was estimated as dLA × LDW/LA. From these data, caterpillar relative growth rate, (RGR, g dw caterpillar. g dw^−1^ caterpillar. day^−1^), was calculated as dCDW/(avgCDW × *t*), where dCDW is the dry weight increase of caterpillars during the entire feeding period, calculated as CDW2 − (CFW1 × CDMC), avgCDW is the average dry weight of caterpillars during the feeding period, calculated as 0.5 × (CDW2 + (CFW1 × CDMC), and *t* is the duration of the bioassay (1 or 2 days for Bio1 and Bio2, respectively). Similarly, relative consumption rate (RCR, g dw leaf eaten. g dw^−1^ caterpillar. Day^−1^) was calculated as dLDW/(avgCDW × *t*). The efficiency of conversion of ingested food (ECI), a measure for how efficient caterpillars can convert leaf material into caterpillar mass, was calculated as ECI = RGR/RCR (Scriber and Slansky, 1981).

### Leaf Chemical Analyses

From each of the plants used in the bioassays, one leaf, viz. the next younger leaf than the one used for the bioassay (i.e., the 2nd and 5th youngest fully expanded leaf for Bio1 and Bio2, respectively), was harvested simultaneously with the bioassay leaf to analyze leaf morphological traits and primary and secondary metabolites. This enabled us to correlate variation in caterpillar growth parameters with variation in leaf morphological and biochemical traits at the individual plant level. After measuring leaf fresh weight leaves were spread out on a glass plate to scan total leaf area using an Epson Perfection V850 pro scanner. Leaf area was estimated from the images using WinFOLIA software (Regent Instruments Inc., Quebec, Canada). Thereafter, leaves were dried for 48 h. in a stove at 40°C to estimate leaf dry weight and calculate specific leaf area (SLA, leaf area per unit leaf dry weight) and leaf dry matter content (LDMC, leaf dry weight divided by leaf fresh weight). Dried leaves were ground to a fine powder using a Retsch mill ball (Retsch GmbH, Haan, Germany). Shoot total nitrogen and carbon concentrations were measured based on 1 mg of ground leaf sample using a Flash EA1112 elemental analyzer (Thermo Fisher Scientific Inc., Waltham, MA, United States). To determine shoot phosphorus concentrations, 3 mg of ground leaf sample was ignited at 550°C for 30 min in a muffle furnace, then extracted with 10 ml of 2.5% potassium persulfate, and measured with an Auto-Analyzer (Seal QuAAtro SFA system; Murphy and Riley, 1962). To determine leaf concentrations of the two IGs aucubin and catalpol, 25 mg of ground leaf sample was extracted by shaking overnight in 10 ml 70% methanol, filtered over Whatman #4 filter paper, readjusted to 10 ml, diluted 10x with milliQ water, and analyzed by HPLC following Marak et al. (2002b) using geniposide as internal standard. Leaf N, P, C, and IG concentrations were expressed as % of leaf dry weight.

### Statistical Analyses

Sequential harvest data (shoot, root, and total dry weight, RMF), were analyzed using generalized linear mixed models (procedure MIXED, SAS v. 9.2, SAS Institute, Cary, NC). The factors Time (T), Light (L), AMF (M), and soil P (P) and their interactions were entered as fixed factors. Block and block × light were entered as random factors. As the experimental set-up followed a split plot design, the whole-plot factor (light) was tested against the whole-plot error term block × light, whereas the other factors were tested against the pooled residual error term. Separate analyses per time point were performed for closer inspection of L, M, and P effects at each of the different time points. Dry weights were log-transformed prior to analysis to meet assumptions of normality of the distribution of residuals and homogeneity of variances. Similar models were used to test effects of light, soil P, and their interaction on root colonization by AMF, for 3- and 6-week old plants, separately.

Bioassay data (RGR, RCR, ECI, SLA, LDMC, and leaf biochemical traits) were also analyzed using generalized linear mixed models with the factors light (L), AMF (M), soil P (P), and jasmonic acid (J) and their interactions as fixed factors and block and block × light as random factors. Leaf IG concentrations were square-root transformed to meet assumptions of normality of the distribution of residuals and homogeneity of variances. In addition, forward stepwise multiple regressions (inclusion/removal criterion *p* < 0.10) were performed to identify leaf traits significantly associated with variation in caterpillar RGR, RCR, and ECI among plants (procedure REG, SAS v. 9.2, SAS Institute, Cary, NC).

## Results

### Effects of AMF, Light, and Soil P on Root Colonization by *Funneliformis mosseae*

**Figure 1:**
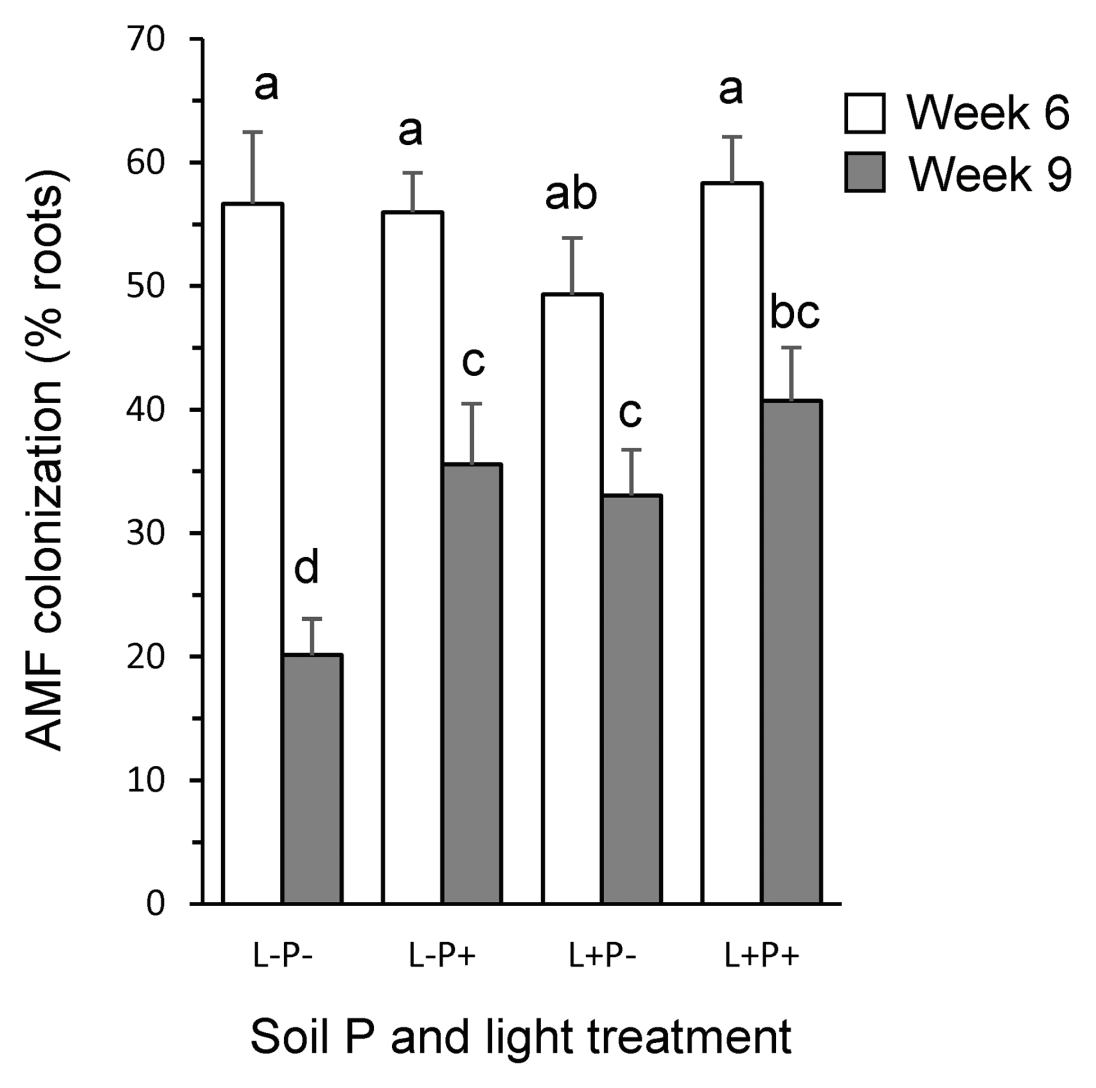
Percent of *P. lanceolata* roots colonized by the mycorrhizal fungus *F. mosseae* for plants grown under four combinations of light intensity (L−: low light; L+: high light) and soil phosphorus treatments (P−: low soil P; P+: high soil P). Open bars: 6 weeks after seedling tansplantation; gray bars: 9 weeks after seedling transplantation. Bars that do not share the same letter are significantly different from each other (*post hoc* tests using LS means, *p* < 0.05).

Root colonization by *F. mosseae* after 6 weeks of growth was high (55.0%) and independent of light or soil phosphorus treatment (Figure 1; Supplementary Table S1). After 9 weeks of growth, root colonization levels were overall much lower (32.4%), and, contrary to expectation, were significantly higher under high soil P (38.1%) than under low soil P (26.6%; *p* < 0.05) but, as expected, marginally lower under low light (27.8%) than under high light conditions (36.9%; *p* = 0.058). At both points in time, no vesicles and only few arbuscules were observed (1.7 and 2.4% in weeks 6 and 9, respectively), independent of light, soil P, or their interaction (all *p* > 0.10). No colonization was observed in non-inoculated plants.

### Effects of AMF, Light, and Soil P on Root and Shoot Biomass

**Figure 2:**
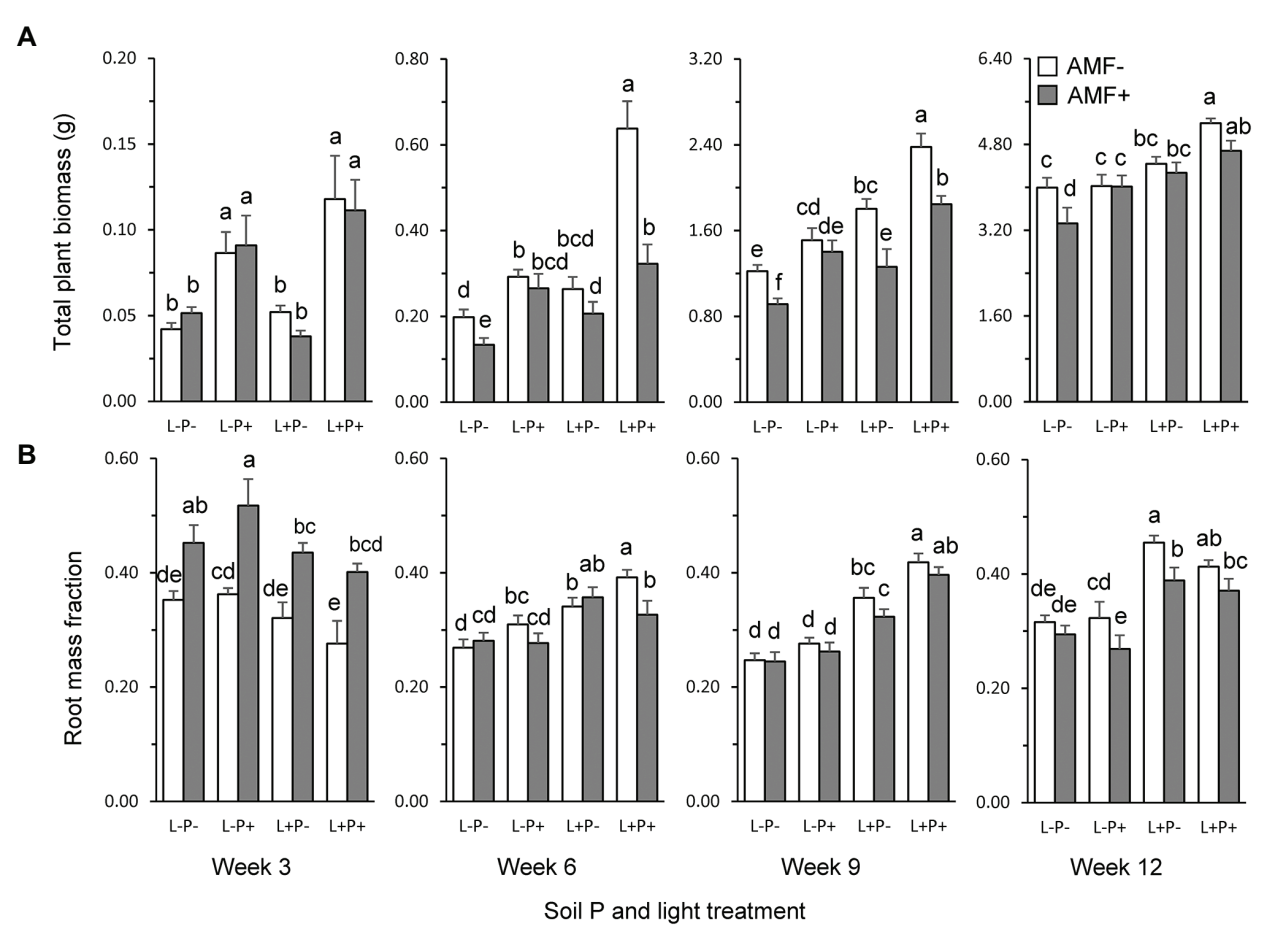
**(A)** Total plant dry weight, and **(B)** RMF (root biomass/total biomass) of *P. lanceolata* plants at four time points during growth (panels from left to right: 3, 6, 9, and 12 weeks after seedling transplantation). Each panel displays results for plants grown under four different combinations of light intensity (L-: low light; L+: high light) and soil phosphorus treatment (P-: low soil P; P+: high soil P). Open bars: non-mycorrhizal plants; gray bars: plants inoculated with the AMF *F. mosseae*. Bars within panels that do not share the same letter are significantly different from each other (*post hoc* tests using LS means, *p* < 0.05).

Total dry weight of plants was overall enhanced by soil P and light, but reduced by the presence of AMF (Figure 2A; Table 1). The strength of these effects varied over time (Table 1: TxL, *p* < 0.05; TxP, *p* < 0.001; TxM, *p* < 0.01). The MGR (the percent difference in plant dry weight of AMF plants compared to non-mycorrhizal plants), averaged across light and soil P treatments, was significantly negative from week 6, when the growth reduction was strongest (33%), but the extent of this reduction declined afterward (Supplementary Table S2). By contrast, high soil P enhanced total dry weight, but the extent of this enhancement also steadily declined over time (Figure 2A, averaged across light and AMF treatments: +122, +89, + 37, and +12%, in weeks 3, 6, 9, and 12, respectively). Similarly, high light enhanced total dry weight from week 9, (Figure 2A), and the extent of enhancement declined from +61% to +45 and +21% in weeks 6, 9, and 12, respectively.

**Table 1:** General Linear Mixed Models of the effects of time (T), light intensity (L), soil phosphorus (P), and Arbuscular Mycorrhizal Fungi (AMF; M), on growth and morphological traits of *Plantago lanceolata:* total, shoot and root dry weight and root mass fraction (RMF, root weight/total weight).

Source	df	Total DW	Shoot DW	Root DW	RMF
		*F*	*F*	*F*	*F*
T	3, 138	0.5^***^	2623.7^***^	1601.8^***^	25.0^***^
L	1, 11	50.6^***^	21.9^***^	100.6^***^	67.7^***^
P	1, 138	148.6^***^	135.4^***^	109.5^***^	2.6
M	1, 138	27.1^***^	33.1^***^	14.3^***^	1.9
TxL	3, 138	3.5^*^	2.1^+^	15.7^***^	33.7^***^
TxP	3, 138	14.4^***^	13.5^***^	10.9^***^	3.9^*^
TxM	3, 138	4.4^**^	3.6^*^	12.3^***^	29.1^***^
LxP	1, 138	2.9^+^	4.5^*^	0.6	1.6
LxM	1, 138	4.1^*^	2.4	2.6	0.9
PxM	1, 138	0.4	0.5	0.0	0.3
TxLxP	3, 138	0.8	2.0	0.1	3.0*
TxLxM	3, 138	0.8	0.7	0.4	0.0
TxPxM	3, 138	0.6	0.4	1.2	1.7
LxPxM	1, 138	0.8	0.6	0.3	0.0
TxLxPxM	3, 138	3.2^*^	3.7^*^	2.5^+^	0.8

Time represents the age of plants, 3, 6, 9, or 12 weeks after seedling transplantation. AMF represents inoculation with Funneliformis mosseae. Values are *F*-values. Values in bold indicate significant effects (p < 0.05).

+ *p* < 0.10.

* *p* < 0.05.

** *p* < 0.01.

*** *p* < 0.001.

df, degrees of freedom; denominator degrees of freedom are 11 for effects of light and 138 for the other factors. RMF, Root mass fraction.

Contrary to expectation, AMF inoculation was not least beneficial for plant dry weight production under conditions considered most unfavorable for trade, i.e., low light and high soil P (Figure 2A). In fact, the combination of low light and high P was the only condition under which we did not observe a negative, but an overall neutral MGR (Supplementary Table S2). The reduction in total biomass by AMF was on average stronger under high than under low light conditions (Table 1: LxM, *p* < 0.05; Figure 2A; Supplementary Table S2). Furthermore, we observed a more complex interaction (Table 1, TxLxPxM, *p* < 0.05) indicating that the interactive effects of light and soil P on the MGR varied over time.

Both shoots and roots contributed to the response of total plant biomass to AMF, but the two plant tissues strongly differed in the temporal dynamics of their responses (Table 1). Overall, shoot biomass was reduced by AMF up to week 9, but no longer at final harvest (Supplementary Figure S1A; Supplementary Table S2). By contrast, root biomass was initially enhanced by AMF, but reduced by AMF from week 6 onwards (Supplementary Figure S1B; Supplementary Table S2). Effects of AMF on the RMF therefore shifted from positive to negative over the course of time (Figure 2B; Table 1: TxM, *p* < 0.001; Supplementary Table S2).

Roots and shoots also differed in the temporal dynamics of their responses to light and soil P (Table 1). Shoot biomass initially responded positively to both soil P and light, but was only enhanced by higher P at final harvest (Supplementary Figure S1A). By contrast, root biomass was initially only enhanced by higher P, but at final harvest only by light availability (Supplementary Figure S1B). As a result, at final harvest, shoot biomass was more strongly determined by soil P, whereas root biomass was more strongly determined by light, i.e., tissue growth was most strongly limited by the resources in the other compartment (Supplementary Figure S1).

### Effects of JA, AMF, Light, and Soil P on Caterpillar Performance

#### First Bioassay

**Figure 3:**
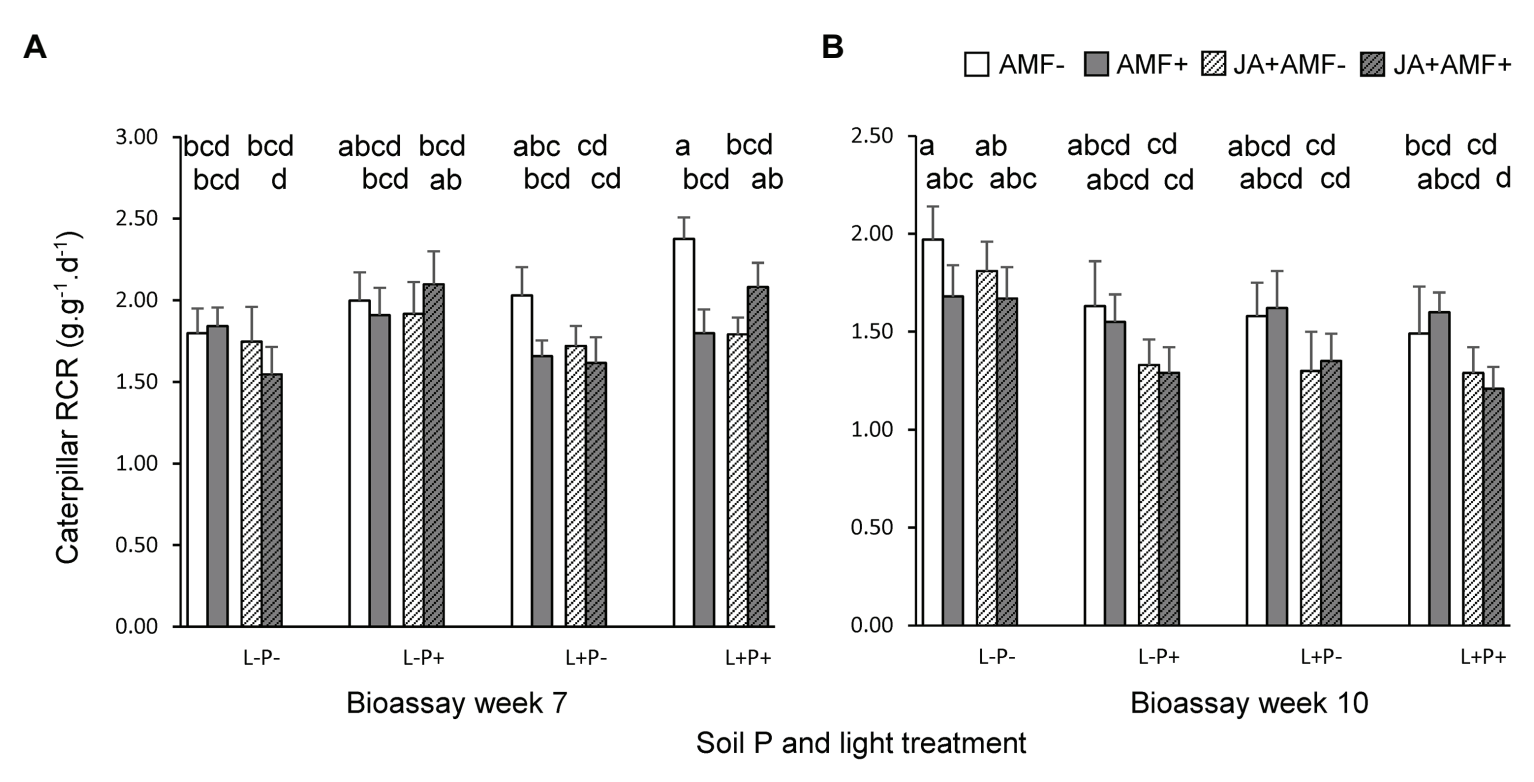
Relative consumption rates (RCRs; amount of leaf mass eaten per unit caterpillar weight per day) of *M. brassicae* caterpillars feeding on *P. lanceolata* during two bioassays, performed when plants were **(A)** 7 weeks old (left panel), and **(B)** 10 weeks old (right panel). Each panel displays results for plants grown under four combinations of light intensity (L-: low light; L+: high light) and soil phosphorus treatments (P-: low soil P; P+: high soil P). In addition, plants had either be challenged by leaf application of jasmonic acid (JA) 48 h. prior to the bioassay (hatched bars) or not (non-hatched bars) and plants had either been inoculated with the AMF *F. mosseae* (bars with gray background) or not (white background). Bars within panels that do not share the same letter are significantly different from each other (*post hoc* tests using LS means, *p* < 0.05). The top row of letters refers to bars that display treatments without AMF, the second row of letters to those with AMF.

In the first bioassay (7-week old plants), application of JA 2 days before the bioassay reduced the RCR of *M. brassicae* caterpillars by on average 6.1% (*p* < 0.05, Table 2; Figure 3A), indicating that there was JA-induced resistance. However, the extent of JA-induced resistance was independent of soil P and light (LxP and LxJ interactions, *p* > 0.4, Table 2). As hypothesized, the effect of JA depended on the presence of AMF (JxM interaction, *p* < 0.05, Table 2), but the direction of this interaction was unexpected. In the absence of AMF, JA significantly decreased RCR by on average 14.0% (*p* = 0.02), whereas in the presence of AMF, JA did not affect the RCR of caterpillars (*p* > 0.6; Figure 3A). Similarly, in the absence of a prior JA-stimulus, AMF reduced caterpillar RCR by 13.5% (*p* = 0.02), indicating AMF-induced resistance, whereas after a prior JA stimulus, AMF did not affect caterpillar RCR (*p* > 0.6). When the interactions between AMF and JA effects were studied separately under each of the four abiotic conditions (i.e., combinations of light and soil P), a significant interaction was only observed under high light and high soil P [F(1,24) = 11.0, *p* = 0.003] and not under the three other treatment combinations. However, the three-way interactions between JA, AMF, and light (Table 2, *p* = 0.08), or between JA, AMF, and soil P (Table 2, *p* = 0.09) were only marginally significant. Therefore, we can only conclude that there is a tendency that the strength of the AMF-induced suppression of the JA response depends on the abiotic conditions, and further studies are needed to support this observation. In conclusion, at the timescale of the experiment, JA-induced resistance was independent of soil P and light, and AMF did not prime plants for a JA-mediated response against *M. brassicae* feeding but, on the contrary, antagonized the JA-response of plants to these caterpillars, and this response tended to by most strongly expressed under high soil P and light conditions.

**Table 2:** General Linear Mixed Models of the effects of light intensity (L), soil phosphorus (P), jasmonic acid treatment (J), and AMF (M), on leaf traits *P. lanceolata* plants and on growth and consumption parameters of *Mamestra brassicae* caterpillars feeding on these plants.

Source	Ndf	Caterpillar traits				Leaf traits					
		RGR	RCR	ECI	Leaf P	Leaf N	Aucubin	Catalpol	SLA	LDMC
		F	F	F	F	F	F	F	F	F
L	1	1.4	0.1	0.3	35.5^***^	17.0^**^	2.4	0.1	6.5^*^	30.7^***^
P	1	2.4	10.0^**^	2.1	95.7^***^	35.2^***^	0.6	6.1^*^	10.5^**^	48.4^***^
J	1	5.0^*^	2.2	0.0	6.2^*^	7.5^**^	1.4	1.9	1.6	0.4
M	1	1.3	1.7	0.7	2.8^+^	2.3	2.5	0.4	14.9^***^	0.7
LxP	1	1.6	0.0	1.0	0.0	1.7	0.7	0.4	0.5	3.8^+^
LxJ	1	0.0	0.4	0.1	0.6	0.1	0.1	0.0	0.6	0.4
PxJ	1	0.0	0.7	1.5	7.7^**^	0.1	1.9	0.6	0.0	0.3
LxM	1	0.2	1.3	0.9	12.4^***^	4.6^*^	0.7	0.0	0.2	3.3^+^
PxM	1	0.0	0.5	0.9	9.8^**^	4.9^*^	0.3	0.4	0.5	6.1^*^
JxM	1	0.1	4.0^*^	4.8^*^	0.1	1.0	0.0	0.0	0.2	0.1
LxPxJ	1	0.1	0.5	0.2	0.0	0.0	1.5	0.0	0.3	0.4
LxPxM	1	1.6	0.0	1.0	1.0	0.0	0.1	0.1	1.6	0.1
LxJxM	1	1.6	3.1^+^	0.0	1.0	0.0	0.3	0.0	0.2	0.0
PxJxM	1	0.3	2.9^+^	0.3	1.0	1.4	0.6	1.8	0.9	2.6
LxPxJxM	1	0.6	0.0	0.7	0.7	0.4	0.1	0.3	0.3	0.9

Data for bioassay 1 (7-week old plants). Values are *F*-values. Values in bold indicate significant effects (p < 0.05).

+ *p* < 0.10.

* *p* < 0.05.

** *p* < 0.01.

*** *p* < 0.001.

Ndf, numerator degrees of freedom. Denominator degrees of freedom (Dnf) are eight for effects of light. Dnf for other factors are: 111 for RGR, 98 for RCR and RCR, 67 for leaf N and P, 112 for leaf aucubin and catalpol, SLA, and LDMC. RGR, relative growth rate; RCR, relative consumption rate; ECI, efficiency of conversion of ingested food; leaf N, leaf nitrogen concentration; leaf P, leaf phosphorus concentration; SLA, specific leaf area; and LDMC, leaf dry matter content (% dry weight).

Caterpillar RCR was also directly affected by soil P levels (Table 2: *p* < 0.01); RCR of caterpillars was on average 12.6% lower on plants grown under low soil P than on under high soil P, indicating that plants grown under low soil P were more resistant to caterpillar feeding. Light did not directly affect caterpillar RCR (Table 2).

The RGR of caterpillars (Supplementary Figure S2A), which is the product of the RCR and the ECI, was only affected by JA application (7.0% reduction, *p* < 0.05, Table 2) and was independent of the presence or absence of AMF. This was due to the fact that the negative effects of AMF on the RCR of caterpillars feeding on plants that had not been treated with JA were partly offset by positive effects of AMF on the efficiency with which these caterpillars could convert the ingested leaf material into caterpillar mass (ECI) under these conditions (Supplementary Figure S2B; Table 2, JxM, *p* < 0.05).

#### Second Bioassay

Results for the second bioassay (10-week old plants) were qualitatively different from those of the first bioassay. Like in the first bioassay, JA application reduced caterpillar RCR, on average by 14.3% (*p* < 0.01, Table 3; Figure 3B), and the extent of JA-induced resistance was independent of soil P and light conditions (Table 3). However, in this bioassay, there was no interaction between JA and AMF effects (Table 3), indicating that the fungus did not prime, nor repress, JA-responses. There were also no interactions between effects of AMF and light or soil P (Table 3). Surprisingly, in contrast to the first bioassay, lower levels of soil P resulted in a significant 13.9% enhancement, not reduction, of caterpillar RCR (Figure 3B; Table 3, *p* < 0.05). Similarly, lower light levels resulted in a 12.5% enhancement of caterpillar RCR (Figure 3B; Table 3, *p* < 0.05).

**Table 3:** General Linear Mixed Models of the effects of light intensity (L), soil phosphorus (P), jasmonic acid treatment (J), and AMF (M), on leaf traits *P. lanceolata* plants and on growth and consumption parameters of *M. brassicae* caterpillars feeding on these plants.

Source	Ndf	Caterpillar traits				Leaf traits					
		RGR	RCR	ECI	Leaf P	Leaf N	Aucubin	Catalpol	SLA	LDMC
		F	F	F	F	F	F	F	F	F
L	1	1.6	5.1^*^	0.4	15.3^**^	48.9^***^	8.0^*^	7.2^*^	100.7^***^	118.1^***^
P	1	0.8	5.8^*^	3.8^+^	22.4^***^	0.5	3.4+	6.3^*^	22.4^***^	10.9^**^
J	1	4.6^*^	8.1^**^	0.3	1.3	0.3	0.0	0.1	3.4^+^	1.1
M	1	0.1	0.5	0.7	66.8^***^	1.0	0.6	1.0	28.7^***^	16.8^***^
LxP	1	1.6	2.6	0.0	1.1	4.3^*^	0.0	1.0	8.3^**^	2.8^+^
LxJ	1	0.1	0.4	0.0	9.0^**^	1.6	0.9	0.3	1.1	0.2
PxJ	1	0.1	0.5	1.3	0.4	0.7	0.5	1.5	0.0	0.5
LxM	1	0.0	1.0	1.2	0.0	0.7	0.3	0.0	0.8	0.0
PxM	1	2.2	0.1	6.0^*^	11.3^**^	7.9^**^	1.5	1.1	9.2^**^	5.4^*^
JxM	1	0.1	0.0	0.2	1.7	0.2	0.0	0.2	0.3	1.7
LxPxJ	1	0.0	0.3	0.3	1.6	0.0	0.4	0.2	3.1^+^	1.6
LxPxM	1	0.2	0.3	0.1	0.3	0.9	0.0	0.3	3.1^+^	0.1
LxJxM	1	0.8	0.3	0.1	6.3^*^	2.2	0.5	0.3	2.7	0.0
PxJxM	1	0.4	0.2	0.0	0.3	0.1	0.1	0.0	0.1	0.0
LxPxJxM	1	1.1	0.0	1.0	1.0	0.3	0.0	0.2	6.8^*^	2.3

Data for bioassay 2 (10-week old plants). Values are *F*-values. Values in bold indicate significant effects (p < 0.05).

+ *p* < 0.10.

* *p* < 0.05.

** *p* < 0.01.

*** *p* < 0.001.

Ndf, numerator degrees of freedom. Denominator degrees of freedom (Dnf) are 11 for effects of light. Dnf for other factors are: 93 for RGR, RCR, and ECI, and 106 for the other traits. RGR, relative growth rate; RCR, relative consumption rate; ECI, efficiency of conversion of ingested food; leaf N, leaf nitrogen concentration; leaf P, leaf phosphorus concentration; SLA, specific leaf area; LDMC, leaf dry matter content (% dry weight).

Like in the first bioassay, caterpillar RGR was only reduced by JA application (Supplementary Figure S2C, 11.5% reduction; Table 3, *p* < 0.05), so effects of light and soil P on RCR did not translate into differences in RGR. AMF enhanced the ECI under low soil P (+17.2%), but not under high soil P (−8.6%; Supplementary Figure S2D), resulting in a significant interaction between AMF and soil P effects on ECI (Table 3, *p* < 0.05).

### Plant Traits Potentially Mediating Treatment Effects on Caterpillar Performance

#### Effects of JA, AMF, Light, and Soil P on Leaf Biochemistry and Morphology

**Figure 4:**
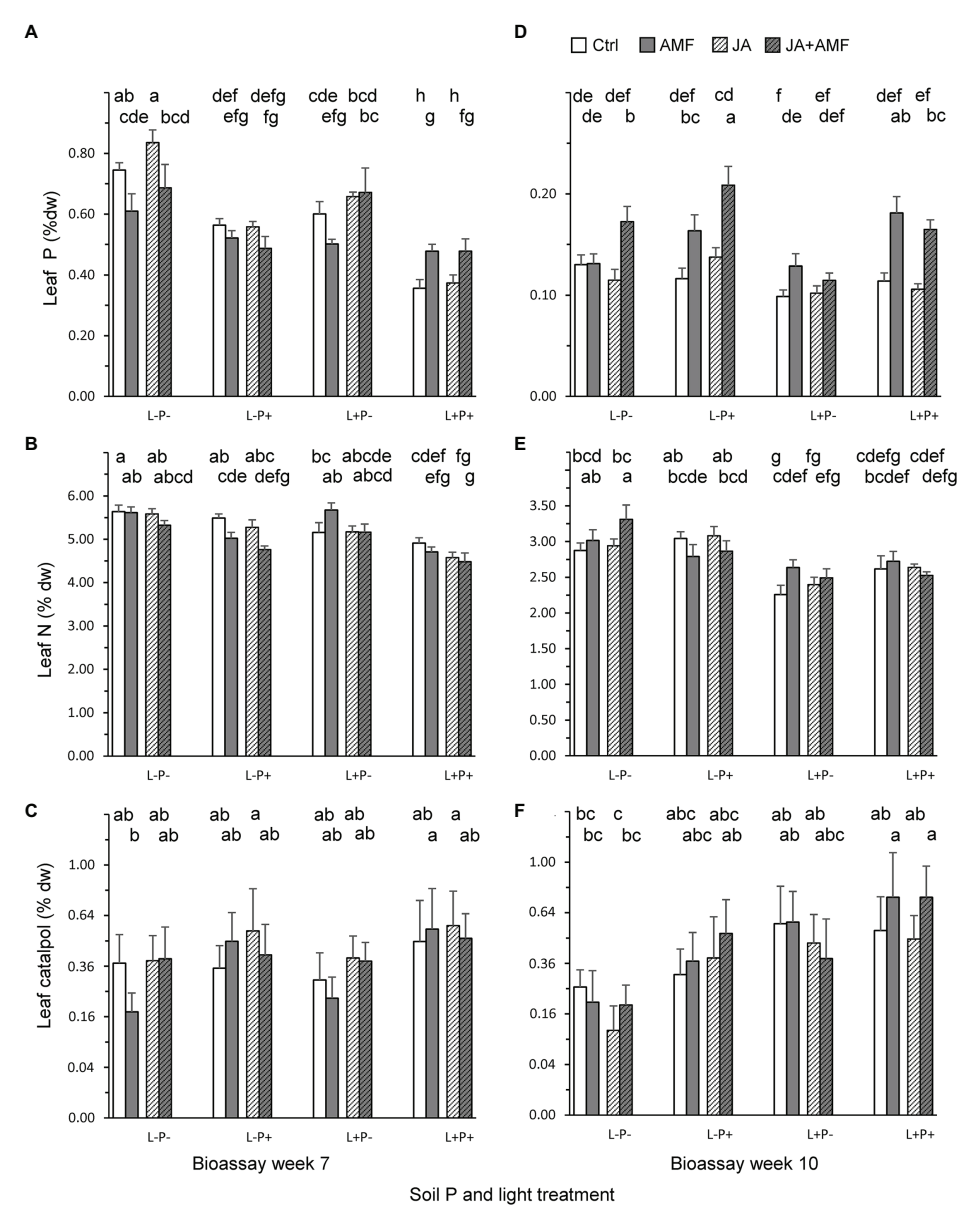
Leaf concentrations of primary and secondary metabolites in *P. lanceolata* plants at the time of two bioassays, when plants were 7 (left panels) and 10 weeks (right panels) old. **(A,D)** Leaf phosphorus; **(B,E)** Leaf nitrogen; **(C,F)** Leaf catalpol. Each panel displays results for plants grown under four combinations of light intensity (L-: low light; L+: high light) and soil phosphorus treatments (P-: low soil P; P+: high soil P). In addition, plants had either be challenged by leaf application of jasmonic acid (JA) 48 h. prior to the bioassay (hatched bars) or not (non-hatched bars) and plants had either been inoculated with the arbuscular mycorrhizal fungus (AMF) *F. mosseae* (bars with gray background) or not (white background). Bars within panels that do not share the same letter are significantly different from each other (*post hoc* tests using LS means, *p* < 0.05). The top row of letters refers to bars that display treatments without AMF, the second row of letters to those with AMF.

Leaf biochemistry was strongly affected by AMF, soil P, and light treatments. In 7-week old plants (Figures 4A–C; Table 2), leaf P and N concentrations were reduced under high light and, surprisingly, also under high soil P conditions (Figures 4A,B; Table 2, all *p* < 0.01). By contrast, AMF enhanced leaf P under high light and high soil P conditions, but reduced leaf P under low light and low soil P conditions, basically buffering variation in leaf P under the various light and soil P conditions (Figure 4A; Table 2: LxM and PxM, *p* < 0.01). Application of JA 3 days before leaf metabolite assessment slightly reduced leaf N (Figure 4B) but enhanced leaf P under low soil P conditions (Figure 4A; Table 2: PxJ, *p* < 0.01). High light and soil P both enhanced LDMC (Supplementary Figure S3A) and reduced SLA (Supplementary Figure S3B), changes that are commonly associated with reductions in leaf palatability. By contrast, AMF enhanced SLA (Supplementary Figure S3B), and had variable effects on LDMC, depending on soil P (Table 2). Unexpectedly, AMF and JA treatments did not affect leaf levels of the defense metabolites catalpol (Figure 4C) or aucubin (Supplementary Figure S3C; Table 2). By contrast, high soil P did increase leaf levels of catalpol (Figure 4C; Table 2).

In 10-week old plants (Figures 4D–F; Table 3), high light conditions continued to reduce leaf N and P concentrations (Figures 4D,E; Table 3, all *p* < 0.01), but high soil P conditions enhanced leaf P, especially in mycorrhizal plants. Indeed, at this later stage of the interaction, AMF enhanced leaf P under a wide range of conditions, but again most strongly under high soil P (Figure 4D; Table 3: PxM, *p* < 0.01). As observed for 7-week old plants, high light and soil P conditions enhanced LDMC and reduced SLA in 10-week old plants as well (Supplementary Figures S3D,E; Table 3: all *p* < 0.001) whereas, conversely, AMF reduced LDMC and enhanced SLA especially under low soil P conditions, both expected to result in higher leaf palatability. As observed in 6-week old plants, AMF did not affect leaf levels of the defense metabolites catalpol or aucubin (Table 3) but high soil P increased levels of catalpol (Figure 4F; Table 3), In addition, high light induced higher levels of both catalpol and aucubin (Figure 4F; Supplementary Figure S3F; Table 3). JA application 4 days prior to leaf metabolite assessment did not induce any changes in the measured metabolites and traits in 10-week old plants (Table 3).

#### Associations Between Plant Traits and Caterpillar Performance

**Figure 5:**
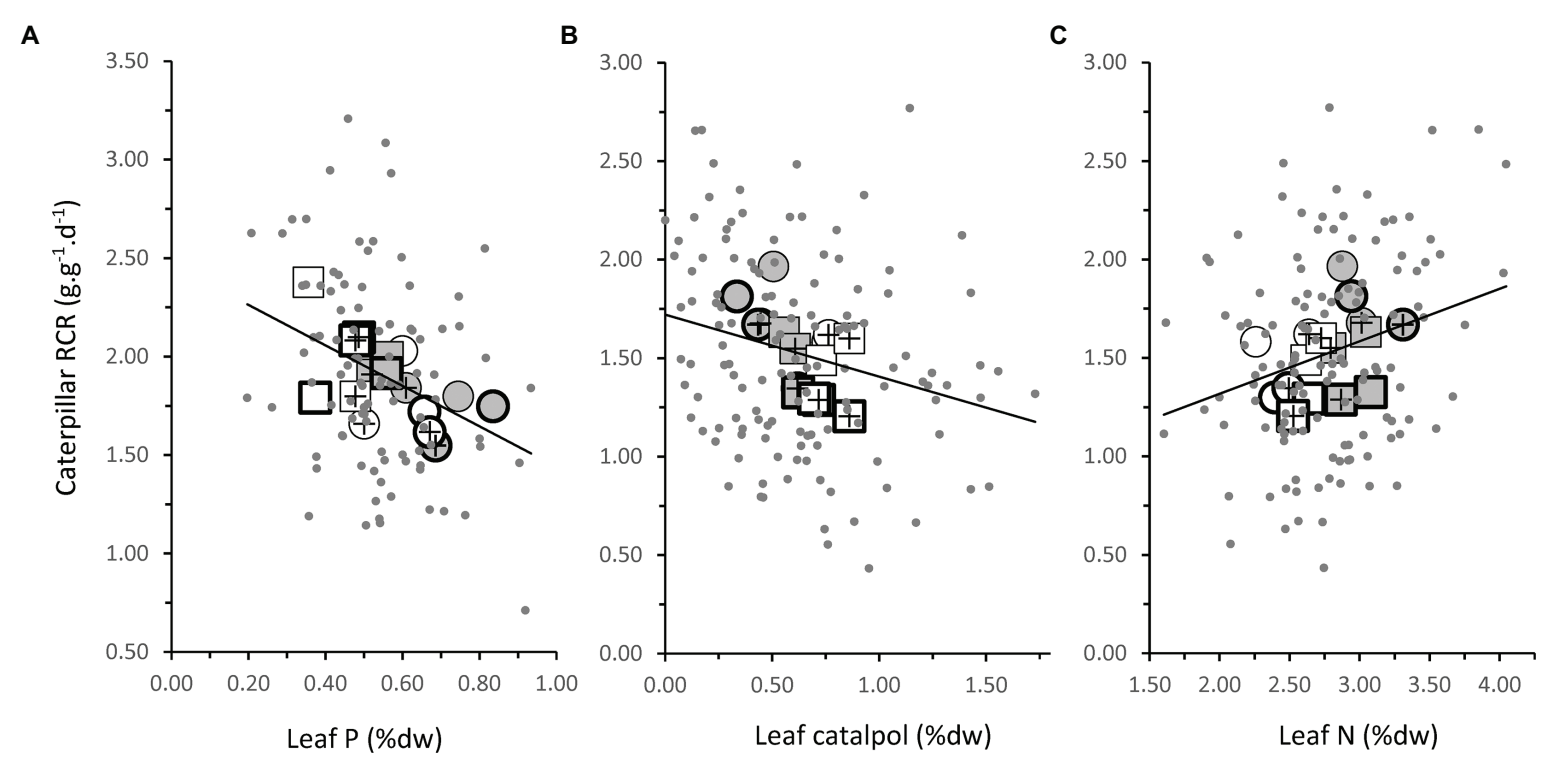
Relationship between leaf traits of *P. lanceolata* plants and the RCR of caterpillars of *M. brassicae* feeding on them. **(A)** Leaf phosphorus concentration at the time of bioassay 1; **(B)** Leaf catalpol at the time of bioassay 2; and **(C)** Leaf nitrogen concentration at the time of bioassay 2. Gray dots represent data points for individual plants. Symbols represent treatment means. Closed symbols: plants grown under low light; open symbols: high light. Circles: plants grown under low soil phosphate; squares: high soil phosphate. Symbols with a plus sign: plants inoculated with AMF; no plus sign: without AMF. Thick symbols: plants challenged by jasmonic acid application 48 h. prior to bioassays, thin symbols: no jasmonic acid application. Lines are slopes from linear regressions (all *p* < 0.05). Note that the values along the leaf catalpol axis are square-root transformed values.

In the first bioassay, leaf traits explained 9.9% of the variation in RCR (Table 4: *p* < 0.01). High RCR was associated with low leaf P (Figure 5A; Table 4: *p* < 0.01). This could partly explain why caterpillar RCR was enhanced by the high soil P treatment in the first bioassay, which resulted in a reduction of leaf P concentrations by on average 27.8% (Figure 4A). Leaf P was also the only leaf trait explaining a significant proportion of the variation in RGR (3.2%, *p* < 0.05); higher RGR was associated with lower leaf P. None of the leaf traits could explain a significant part of variation in ECI (5.1%, *p* = 0.09).

**Table 4:** Forward multiple regressions of *P. lanceolata* leaf traits on growth and consumption parameters of caterpillars of *M. brassicae* feeding on these plants.

Source	Bioassay 1			Bioassay 2		
	RGR	RCR	ECI	RGR	RCR	ECI
Specific leaf area (SLA)	**-**	**-**	**-**	**-**	**-**	**-**
Leaf dry matter content (LDMC)	**-**	**-**	**-**	**-**	**-**	**-**
Leaf N concentration	**-**	**-**	**-**	**-**	+0.21^**^	**-**
Leaf P concentration	−0.22^*^	−0.31^**^	**+**0.16	**-**	**-**	**-**
Leaf aucubin concentration	**-**	**-**	**-**	**-**	**-**	**-**
Leaf catalpol concentration	**-**	**-**	**-**	−0.18^*^	−0.20^*^	**-**
Model *R*^2^ (%)	4.7	9.9	2.6	3.2	10.2	**-**
F-value	4.6^*^	10.2^**^	2.5	4.3^*^	7.3^***^	**-**
df	1,93	1,93	1,93	1,129	2,128	**-**

Values are standard partial regression coefficients. Data for bioassays 1 and 2 (7- and 10-week old plants, respectively). Model fit (100 × *R*^2^), *F*-tests, and their degrees of freedom (df) are indicated at the bottom. Values in bold indicate significant effects (p < 0.05).

* *p* < 0.05.

** *p* < 0.01.

*** *p* < 0.001.

RGR, relative growth rate; RCR, relative consumption rate; and ECI, efficiency of conversion of ingested food.

In the second bioassay, leaf traits explained 10.2% of the variation in RCR (Table 4: *p* < 0.01). High RCR was associated with low leaf concentrations of the defense chemical catalpol (Figure 5B; Table 4: *p* < 0.05) and high leaf N (Figure 5C; Table 4: *p* < 0.01). This could partly explain why caterpillar RCR was reduced by high light and high soil P conditions, which resulted in increases in leaf catalpol levels by on average 43.7 and 80.2%, respectively (Figure 4F). In addition, the 15.2% lower levels of leaf *N* under high light conditions (Figure 4E) could have contributed to the lower caterpillar RCR under these conditions. Leaf traits did not explain a significant proportion of the variation in ECI in the second bioassay and only 3.5% of the variation in RGR (Table 4: *p* < 0.05). As observed for RCR, high RGR was associated with low leaf concentrations of catalpol (Table 4). JA application did not affect leaf N, or leaf catalpol in the second bioassay, hence JA-induced resistance to *M. brassicae* was mediated by plant traits not measured in the current experiment.

Strikingly, SLA and LDMC, that are often strongly associated with insect performance on host plants (Schoonhoven et al., 2005), were strongly affected by light, soil P, and AMF (Supplementary Figure S3), but played no significant role in explaining variation in caterpillar relative consumption or growth rates (Table 4), and therefore did not appear to mediate effects of AMF, light, or soil P on caterpillar performance.

## Discussion

Three main conclusions arise from our study. First, The MGR varied significantly among abiotic conditions and plant ontogenetic stages. However, AMF did not benefit plant growth under any of these abiotic conditions and, contrary to our hypothesis, the association with AMF was not least beneficial for plant growth under a combination of low light and high soil phosphorus conditions, that is usually considered least optimal for trade between the two symbionts. Second, in both of the bioassays, JA induced resistance, but the extent of this induction was independent of light and soil phosphorus conditions. Moreover, AMF inoculation did not prime plants for a stronger jasmonic acid-mediated defense response. On the contrary, as indicated by the caterpillar leaf consumption rates, jasmonic acid application induced resistance in non-mycorrhizal plants, but not in mycorrhizal plants during the first bioassay. This suggests that AMF inoculation suppressed rather than primed the JA-induced defense response of plants at this stage. This modulation of the JA-induced defense response by AMF was only observed under conditions of high resource supply (high light and soil P). However, since the impact of abiotic factors on this interaction was statistically only marginally significant, evidence for significant modulation of the impact of AMF on plant resistance by soil P or light in this system awaits further study. Third, in this experiment, AMF did not affect leaf concentrations of an important class of defense metabolites in *P. lanceolata*, the iridoid glycosides aucubin and catalpol, under any of the soil P or light conditions. AMF inoculation did affect many other leaf traits, including leaf N and P, SLA and leaf dry matter content in a light-, soil P-, and plant age-specific manner. But in spite of these induced changes, AMF did not affect caterpillar consumption and growth other than through suppression of the JA-priming response in younger plants. By contrast, high light and soil P levels reduced the RCR of caterpillars feeding on older plants and these effects were associated with a reduction in leaf nitrogen concentrations and an increase in the leaf concentrations of the defense chemical catalpol in plants grown under these conditions. Abiotic factors were thus important determinants of plant resistance to insect feeding, but these effects appeared to be more strongly mediated by modulating leaf quality than by modulating AMF-induced resistance.

### Effects of AMF, Light, and Soil P on Growth and Biomass Allocation

Functionality of the mycorrhizal symbiosis generally depends on its abiotic and biotic context and consequently, the outcome of plant-AMF interactions can range from mutualism to parasitism (Hoeksema et al., 2010). In our study, inoculation of *P. lanceolata* plants with a strain of the AM fungus *F. mosseae* overall reduced the total biomass of *P. lanceolata* plants, i.e., the MGR was overall negative. This may be related to the fact that we observed substantial rates of root colonization by the fungus, but a relatively low incidence of arbuscules (around 2%) under all environmental conditions tested, indicating a limited functionality of the symbiosis in terms of P-for-C trade. Similar negative MGR values have been reported in *P. lanceolata* following inoculation with another strain of *G. mosseae* (Wang et al., 2015). However, in general, effects of AMF inoculation in *P. lanceolata* have been shown to range from negative to positive (e.g., Ayres et al., 2006; Zaller et al., 2011; Karasawa et al., 2012; Pankoke et al., 2015).

The extent of growth reduction by AMF strongly varied with the age of the plants. However, the effects of light and soil P on MGR were inconsistent over time and generally did not follow predictions from stoichiometry- and trade-based principles (Hoeksema et al., 2010; Johnson, 2010). In particular, MGR did not appear to be lowest under conditions considered to be least optimal for symbiotic trade (low light and high soil P). In fact, this was the only condition under which MGR was consistently neutral during the entire growth period. The absence of a consistent reduction in MGR under high soil P and low light conditions contradicts most other published studies (Johnson, 2010; Konvalinkova and Jansa, 2016). For instance, MGR can shift from positive to neutral at higher soil P (Hetrick et al., 1983) and studies on effect of light availability generally show that the growth benefit of AMF is reduced or becomes negative under shading conditions (Konvalinkova and Jansa, 2016). However, closer inspection of the review by Konvalinkova and Jansa (2016) reveals an interesting pattern. In host-AMF combinations for which an overall positive MGR is observed, shading generally results in a reduction of the MGR. However, in host-AMF combinations for which an overall negative MGR is observed, shading generally does not further reduce MGR (see e.g., Reinhard et al., 1993; Olsson et al., 2010; Grman, 2012). The mechanism underlying the lack of shade-induced reductions in MGR in plants experiencing negative MGR is unknown, but it could simply reflect the fact that whereas all plants experience their lowest MGR under low light conditions, some plant species benefit from AMF under more favorable light conditions, whereas others fail to benefit, resulting in an apparent lack of light responsiveness of MGR. It is unknown whether a similar pattern occurs in plants grown under high soil P.

Growth depressions (negative MGR) have traditionally been viewed as the result of excessive carbon use by AMF, i.e., high allocation of carbon to the fungus relative to the available C-resources under conditions where carbon resources are scarce relative to nutrients, such as under shading and high soil P conditions (Johnson, 2010). Since the discovery that the extent of negative MGR does not necessarily increase with the amount of root colonization or C-drain by the fungus (Li et al., 2008; Grace et al., 2009), a second explanation has been put forward based on nutrient limitation (Smith and Smith, 2011). AM plants can take up nutrients through two different pathways. One is the direct pathway by which plants acquire nutrients from the rhizosphere through their own roots. The other is the mycorrhizal pathway in which the AM fungal partner transfers nutrients to the plant taken up through the extra-radical mycelial network. AMF suppress uptake of P by the plant through its direct pathway. Hence, negative MGR may not only occur in cases of excessive fungal carbon demand, but also when in AM plants the delivery of P through the mycorrhizal is functional, but insufficient to compensate for the reduced uptake of P through the plant’s direct pathway (Smith and Smith, 2012). In those cases, AM plants may suffer P-deficiency resulting in growth depression, for instance under low light conditions when only low amounts of assimilates are available for the fungus. Our experiments were not designed to discriminate between these two mechanisms. However, it is interesting that, despite negative MGR values, leaf P concentrations were still similar or higher in mycorrhizal compared to non-mycorrhizal plants. In seven-week old plants, leaf P concentrations were on average similar between mycorrhizal and non-mycorrhizal plants, although they were less responsive to variation in light and soil P in mycorrhizal plants. In 10-week old plants, leaf P concentrations were even higher in mycorrhizal plants than in non-mycorrhizal plants, despite negative MGR. This suggests that the negative MGR in our study was unlikely to be caused by P-deficiency due to a lack of compensation for reduced P acquisition *via* the plant’s own direct P-uptake pathway by mycorrhizal P delivery.

The extent of the negative MGR diminished over time as plants grew older. At final harvest, MGR was overall close to zero and only remained significantly negative under the lowest resource condition (low light and low soil P). The decline in the extent of negative MGR was accompanied by a decline in AMF root colonization over time, indicating that the production of new fungal structures did not keep up with root growth and the turnover of existing structures. However, the decline in colonization rate was not strongest under the conditions predicted to be least optimal for trade. Instead, the decline was strongest under the lowest resource supply condition (low light, low soil P; 2.8 fold) and weakest under the highest resource supply condition (high light, high soil P; 1.4 fold). As the RGRs of roots under these conditions were similar, this might indicate that there was differential regulation of the AMF by the plant’s complex feedback mechanism known as AOM under these conditions (Vierheilig et al., 2000; Nagy et al., 2009; Mueller and Harrison, 2019). However, despite the fact that the decline in colonization rate was strongest under the lowest resource condition, a negative MGR still persisted under these conditions at final harvest. This indicates that the AMF association was maintained even under the least beneficial conditions. This is a well-known phenomenon. For instance, AMF are usually not eliminated from roots even under very low light intensities (Schubert et al., 1992; Olsson et al., 2010), which has led to the speculation that root colonization by AMF is maintained as an investment for potentially more beneficial trade in the future (Landis and Fraser, 2008; Walder and van der Heijden, 2015; Konvalinkova and Jansa, 2016). Similarly, AMF in common mycorrhizal networks preferentially allocate nutrients to non-shaded plants, but still supply shaded hosts with nutrients and maintain a high colonization in these plants, which has led to the speculation that AMF maintain association with less rewarding hosts to retain “bargaining power” (Fellbaum et al., 2014; Zheng et al., 2015).

### Effects of JA, AMF, Light, and Soil P on Defense Induction and Priming

#### Induction

Unexpectedly, AMF inoculation did not result in the induction of the most commonly studied defense metabolites in *P. lanceolata*, the iridoid glycosides aucubin and catalpol, under any of the experimental conditions. Induction in response to AMF has been commonly reported for catalpol (Gange and West, 1994; Schweiger et al., 2014a; Wang et al., 2015). However, induction of these compounds by AMF has also been shown to vary among AM fungal species. In a single experiment, Bennett et al. (2009) showed that among three fungal species tested, induction of IGs ranged from non-significant by *Glomus* “white” to a significant 1.4 and 3.5 fold increases by *Archaeospora trappei* and *Scutellospora calospora*, respectively. Also other studies have reported no effect of AMF inoculation on leaf IG concentrations in *P. lanceolata* (Fontana et al., 2009).

Since IGs were not induced by AMF inoculation in our study, the question to what extent the induction of these defense chemicals by AMF is dependent on light and soil P conditions awaits future studies. We could only test how the constitutive production of these metabolites varied with light and soil P conditions. Based on stoichiometric principles, production of C-based secondary metabolites such as IGs is expected to be reduced under low light, high nutrient conditions (Fajer et al., 1992). Our study showed that in older plants the production of both aucubin and catalpol was indeed reduced under low light conditions, in agreement with earlier studies (Tamura, 2001; Miehe-Steier et al., 2015). However, in contrast to earlier studies that confirmed a lower production of IGs under high nutrient conditions (Fajer et al., 1992; Darrow and Bowers, 1999; Jarzomski et al., 2000; Marak et al., 2003; Pankoke et al., 2015), we observed higher levels of catalpol under high soil P conditions. It is unknown whether the effect of nutrient limitation on IG production depends on the type of nutrient limitation. As in many of these studies it is unknown what nutrient was limiting growth, it is possible that most of them assessed effects of N- rather than P-limitation that we studied. Further, it should be noted that effect of nutrient limitation on the constitutive production of IGs is not necessarily a good predictor of its effect on the induction of these defense chemicals. For instance, Darrow and Bowers (1999) showed that constitutive levels of IGs were lower at high nutrient level, but the extent of induction by herbivores was independent of nutrient level.

#### Priming

Application of JA to systemic leaves, 24 h prior to the bioassays, significantly reduced the RCRs of caterpillar, during both bioassays. This indicates that plants experienced JA-induced resistance to *M. brassicae*. However, contrary to predictions based on the observation that activation of the PSR can co-activate JA-dependent immunity (Khan et al., 2016; Castrillo et al., 2017); the observed JA-induced resistance was independent of soil P. Our experiments do not allow us to disentangle whether this was due to a lack of induction of PSR or lack of co-activation of JA-dependent resistance. Importantly, our results indicate that, at the timescale studied in the experiment, AMF did not prime plants for a JA-mediated defense response against *M. brassicae* feeding. On the contrary, AMF antagonized the JA-mediated defense response during the first bioassays. In the absence of AMF inoculation, JA significantly reduced the RCR of caterpillars. But whereas we expected an even stronger reduction in the presence of AMF in the case of priming, we actually observed that JA application did no longer reduce caterpillar RCR in plants inoculated with AMF. This raises the question why we did not observe mycorrhizal priming for enhanced defense in our study. Priming is a dynamic process and assessing priming effects at only one point in time, in our case 48 h after challenge, may not capture the event (Martinez-Medina et al., 2016), or the concentration of JA applied may not have been appropriate. However, it is also possible that priming truly did not occur in the study system of *P. lanceolata* and the particular strain of *F. mosseae* that we used, under the environmental conditions that we tested. Despite convincing evidence for mycorrhizal priming in study systems such as tomato (Song et al., 2013), variable results have been reported in others. For instance, Minton et al. (2016) tested mycorrhizal priming of defense in response to the chewing herbivore *Manduca sexta* in two wild *Solanum* species following JA-application to plants that had or had not been pre-inoculated with the AM fungus *Rhizophagus irregularis*. In both plant species JA reduced caterpillar mass, but AMF did not induce, nor prime plant defenses in terms of reduced caterpillar weight gain in response to JA application. Interestingly, at the underlying molecular level, the AM fungus did modulate JA-induced gene expression. But whereas it primed the JA-induced transcription of one of the genes for protein-based chemical defenses (peroxidase), it repressed another (polyphenol oxidase) and did not affect the JA-induced transcriptional response to a third one (proteinase inhibitor) in *Solanum dulcamara*. Moreover, it did not affect any of these in the annual *Solanum ptycanthum*. This indicates, first, that modulation of the JA-induced responses by a single AM strain can differ among plant species and, second, that modulation of JA-responses by AMF at the transcriptional level may range from priming to repression and that it may not translate into defense responses at the phenotypic level.

Our results are in agreement with several earlier studies showing that AMF might not only fail to prime plants for defense but even repress the plant’s defense response to herbivory. Barber (2013) showed that inoculation of cucumber with the AM fungus *R. irregularis* did not affect the amount of leaf feeding by the generalist leaf chewing insect herbivore *Spodoptera exigua* in the absence of prior damage, and actually enhanced feeding after prior damage. Earlier studies in *P. lanceolata* have also yielded mixed results. Wang et al. (2015) showed that in plants that were not inoculated with AMF, the RGR of caterpillars of *S. exigua* decreased with time after prior herbivory by conspecifics, indicating a progressive build-up of induced defense. Inoculation of plants with the AM fungus *F. mosseae* also decreased caterpillar RGR, indicating AMF-induced resistance. However, in plants inoculated with *F.mosseae,* no further induction of defense occurred in response to herbivory, suggesting that AMF did not prime, but suppress, the induction of defense in response to herbivory. This nicely matched with the build-up of one of the defense chemicals, the iridoid glycoside catalpol, in response to previous herbivory in non-inoculated plants, but not in plants inoculated by *F. mosseae*. A very similar response had been observed by Bennett et al. (2009) for the AM fungus *Scutellospora calospora*. In the absence of previous herbivory, this AM fungus induced iridoid glycosides in *P. lanceolata* plants. In non-mycorrhizal plants, previous herbivory by the specialist leaf chewing herbivore *Junonia coenia* also induced iridoid glycosides. However, in plants inoculated with the AM fungus, no further increase in leaf concentrations of these defense chemicals were observed in response to previous herbivory. This suggests that the AM fungus itself induced defense, but that it suppressed defense induction by herbivory. These results could suggest that the processes of induction and priming are interconnected, and that plants already expressing induced defenses in response to AMF are less prone to activate priming (ISR), but by contrast may suppress further induction. However, this is not supported by the pattern observed for other AMF tested by Bennett et al. (2009). For instance, even though the AMF strain “*Glomus white*” itself did not induce iridoid glycosides in *P. lanceolata*, it completely suppressed the strong induction of iridoid glycosides in response to herbivory that was observed in non-mycorrhizal plants (Bennett et al., 2009), like we observed in our study. These studies indicate that whereas priming of defenses may occur in some study systems, opposite patterns may be found in others and our study is not an exception. This may be one of the reasons that a growing number of studies is observing AMF-induced susceptibility (Bernaola et al., 2018; Frew et al., 2018; Real-Santillán et al., 2019) even against insect herbivores that are assumed to be signalled through JA, and calls for future studies examining factors that underlie variation in the extent to which AMF prime their host plants for defense or not.

Our expectation was that the extent to which AMF prime plants for enhanced JA-mediated defense would be affected by light and soil P conditions for several reasons. Firstly, soil phosphate and light are expected to impact AMF colonization rates and functionality. Secondly, there are strong interconnections between the regulation of the PSR and immunity. Finally, the production of defense chemicals following the activation of downstream defense genes will eventually require the availability of sufficient amounts of the resources needed. We observed that the modulation of the JA-induced defense response by AMF during the first bioassay indeed only occurred under one combination of environmental conditions, viz. under the highest resource supply (high light and soil P). Although this suggests that the ability of AMF to modulate the plant’s response to a JA challenge indeed depends on soil P and light, statistical analyses indicated that this dependence on abiotic conditions was only marginally significant. Further studies are therefore required to confirm this environmental dependence. In any case, the results suggest that the AMF-induced suppression of defense activation in response to herbivory was unlikely to be driven by low availability of carbon or nutrient resources, as it did not specifically occur under low resource conditions. The AMF colonization data show that the highest level of AMF proliferation was maintained under high resource conditions. This might suggest that the stronger AMF-induced suppression of defense activation under high resource conditions could be related to higher levels of colonization under these conditions. However, at the time of the first bioassay, when these effects were observed, AMF colonization levels were still overall high, making this explanation less likely.

### Leaf Traits Mediating Effects of JA, AMF, Light, and Soil P on Defense

Two leaf traits were major determinants of caterpillar relative leaf consumption rates in older plants. Leaf consumption rates were positively correlated with leaf N and negatively with leaf catalpol concentrations. These results are in line with a plethora of studies showing that caterpillars of generalist herbivores preferentially feed on leaves with a high nutritional value (low C/N ratio) and low levels of defense metabolites (e.g., Coley et al., 2006). This may explain why caterpillars had higher leaf consumption rates on plants grown under low light and low soil P during the second bioassay. As commonly observed (Poorter et al., 2019), low light conditions resulted in a larger proportion of biomass allocated to leaves (lower RMF), a larger leaf area per unit leaf mass (SLA), higher leaf concentrations of N and P, and in a lower leaf dry matter content (LDMC) and lower leaf concentrations of the C-based defense metabolites aucubin and catalpol in these plants. Similarly, low soil P conditions resulted in increased leaf N and lower leaf catalpol concentrations in older plants. Thus, these two leaf traits may well have mediated the higher susceptibility of plants to caterpillar feeding when grown under low light and low soil P. Traits mediating the lower consumption rates on plants that had been treated with JA could not be identified. Clearly, this was not mediated by increases in the measured defense metabolites aucubin or catalpol. Although the first committed steps in the biosynthesis of these iridoid glycosides can be upregulated in response to JA (Biere, unpublished results), JA application in our experiment did not result in higher levels of these defense metabolites 3–4 days after treatment, in agreement with observations from previous experiments (Sutter and Mueller, 2011; Schweiger et al., 2014b).

Interestingly, AMF strengthened some of the leaf responses to low light in older plants including the increases in SLA and leaf P and decreases in RMF and LDMC. These results corroborate findings from earlier studies showing that under light deprivation AMF can enhance adaptation to low light conditions by increasing SLA and reducing RMF (Konvalinkova et al., 2015), as commonly observed in AM plants (Pankoke et al., 2015; Tomczak et al., 2016). However, unlike light deprivation, AMF did not enhance leaf N, or reduce leaf catalpol concentrations in older plants, the only two traits that affected caterpillar consumption rates in older plants in our study. This explains why, unlike light and soil P, AMF did not directly affect plant susceptibility to caterpillar feeding in our experiments.

Although AMF inoculation did not affect the leaf consumption rates of caterpillars on older plants, it did affect another aspect of caterpillar performance. AMF increased the caterpillar’s efficiency of converting ingested food into caterpillar biomass (ECI) under low, but not under high soil P conditions, i.e., AMF increased leaf quality for caterpillars under low soil P conditions. Interestingly, exactly under these conditions AMF enhanced SLA and reduced LDMC (the latter indicating increased leaf water content). Increases in SLA and leaf water content commonly enhance performance of insect herbivores (Schoonhoven et al., 2005). Furthermore, studies in the congener *Plantago major* revealed that inoculation with the AM fungus *R. irregularis* similarly resulted in a higher SLA and water content (Tomczak et al., 2016) and in an increase in biomass of caterpillars of *M. brassicae* feeding on mycorrhizal plants. Therefore, we hypothesized that modulation of these traits by AMF under low soil P could offer an explanation for the AMF-induced increase in caterpillar ECI under these conditions. However, in our analyses of trait associations with caterpillar performance, we could not show a significant impact of either SLA or LDMC on ECI, hence other, unmeasured, traits may have been responsible for this AMF-induced effect. Also, in our study, the positive effect of AMF on ECI did not translate into a positive effect on caterpillar RGR, indicating that the positive effect on ECI was partly offset by a reduced consumption rate.

The traits explaining effects of treatments on caterpillar consumption rates in young plants differed from those in older plants. In contrast to older plants, low soil P in younger plants was associated with lower caterpillar leaf consumption rates. However, in younger plants, this was not mediated by increased leaf N, or by reduced leaf catalpol concentrations under these conditions. Surprisingly, and in contrast to older plants, younger non-mycorrhizal plants grown under low P showed elevated leaf P concentrations, a response that was buffered in mycorrhizal plants, and high leaf P was associated with lower leaf consumption rates. This result is currently unexplained, but might indicate a highly activated PSR associated with enhanced immunity in younger plants. In general, our results depict a very dynamic interplay of trait modulation by AMF and environmental factors. The effects and relative importance of the different abiotic and biotic conditions that act as modulators of leaf morphological and biochemical traits involved in herbivore resistance varied over the course of time and stage of the plant-AMF symbiosis. This further adds to temporal variation in plant resistance on top of the already present common ontogenetic changes in resistance strategies during ontogeny (Van Dam et al., 2001; Boege and Marquis, 2005; Barton and Koricheva, 2010; Miller et al., 2014).

### Conclusion

We did not find support for our hypothesis that the benefits of AMF for plant growth are reduced under conditions considered unfavorable for trade, i.e., high soil P and low light. We speculate that this may be a general pattern for plants already experiencing an overall negative MGR even at higher light and lower soil P conditions. Furthermore, soil P and light affected the plant’s resistance to a leaf chewing insect herbivore, but these effects were more strongly mediated by direct changes in leaf primary and secondary metabolites than by their modulating effects on mycorrhiza-induced resistance. Finally, our study adds to the growing number of studies reporting that priming of JA-dependent defenses by mycorrhizae may occur in some systems, but that repression of JA-dependent defenses may occur in other systems. In our system, such repression seems to be most strongly expressed under high resource (light, soil P) conditions, but significant modulation of repression by environmental factors awaits confirmation by future studies.

## Data Availability Statement

The datasets generated for this study are available on request to the corresponding author. Data are available in the Dryad repository (https://doi.org/10.5061/dryad.wm37pvmmp).

## Author Contributions

AB and LQ conceived and analyzed the experiments and wrote the manuscript. LQ, MW, and AB performed the experiments. All authors contributed to the article and approved the submitted version.

### Conflict of Interest

The authors declare that the research was conducted in the absence of any commercial or financial relationships that could be construed as a potential conflict of interest.
