# Supplementary material for: Interactive Effects of Mycorrhizae, Soil Phosphorus, and Light on Growth and Induction and Priming of Defense in Plantago lanceolata
Source: Front Plant Sci. 2021 Mar 23;12:647372. doi: 10.3389/fpls.2021.647372 (PMC8021950; doi:10.3389/fpls.2021.647372)
Supplement: Supplementary file 1 [file Data_Sheet_1.docx]

**Supplementary Table 1**. General linear model of the effects of light treatment (L), soil phosphorus treatment (P) and their interaction on the proportion of *Plantago lanceolata* roots colonized by the mycorrhizal fungus *Funneliformis mosseae* at two timepoints: three and six weeks after seedling transplantation.

Six weeks Nine weeks

Source ndf ddf F P F P

L 1 2 0.23 0.680 6.03 0.058^+^

P 1 15 1.04 0.323 **9.83** **0.011***

LxP 1 15 1.63 0.221 1.12 0.315

+: P<0.10; *: P<0.05. Ndf = numerator degrees of freedom; Ddf = denominator degrees of freedom. Since 3-week old plant were harvested from 3 blocks and 6-week old plants from 6 blocks, the ddf for the whole-plot factor light were 2 and 5, respectively.

**Supplementary Table 2**. Mycorrhizal Growth Response (MGR) of *Plantago lanceolata* plants grown under high (+) and low (-) light (L) and soil phosphorus (P) conditions.

L-P- L-P+ L+P- L+P+ All

*Total biomass*

- Week 3 +22.4 +5.1 -27.3 -5.6 -2.4

- Week 6 **-32.5** -9.0 -21.7 **-49.4** **-33.3*****

- Week 9 **-25.1** -7.1 **-30.0** **-22.4** **-21.5*****

- Week 12 -**16.7** -0.3 -3.8 -9.9 **-7.7***

*Shoot biomass*

- Week 3 +2.3 -22.4 **-39.9** -18.2 **-20.4***

- Week 6 **-32.9** -4.4 -22.5 **-43.3** **-29.0*****

- Week 9 **-25.2** -6.0 **-27.2** **-19.5** **-19.4*****

- Week 12 -**14.8** -7.7 -8.0 -3.8 -1.1

*Root biomass*

- Week 3 +59.8 +54.3 -0.2 +22.9 **+34.4***

- Week 6 **-31.6** -19.3 -20.0 **-58.8** **-41.3*****

- Week 9 **-24.7** -10.1 **-35.2** **-26.5** **-25.7*****

- Week 12 **-20.9** -17.0 -17.9 -18.8 **-18.6*****

*Root Mass Fraction*

- Week 3 **+28.3** **+42.8** **+35.6** **+45.3** **+38.0*****

- Week 6 +1.1 -10.4 +4.4 **-16.6** -5.4

- Week 9 -1.0 -5.0 -9.4 -5.2 -5.1

- Week 12 -6.8 **-16.9** **-14.5** -10.1 **-12.1****

MGR = difference in biomass produced by mycorrhizal vs. non-mycorrhizal plants, as a percentage of the biomass produced by non-mycorrhizal plants. Values in bold are significantly different from zero, as inferred from the Mycorrhiza effect in generalized linear mixed models (cf. Table 1 in the main text), either based on analyses of all four time points jointly (last column), or on analyses performed separately for the four different time points (first four columns). *: P<0.05; **: P<0.01; ***: P<0.001.





**Supplementary Figure S1. (A)** Shoot dry weight and **(B)** Root dry weight of *Plantago lanceolata* plants at four time points during growth (panels from left to right: 3, 6, 9, and 12 weeks after seedling transplantation). Each panel displays results for plants grown under four different combinations of light intensity (L-: low light; L+: high light) and soil phosphorus treatment (P-: low soil P; P+: high soil P). Open bars: non-mycorrhizal plants; grey bars: plants inoculated with the arbuscular mycorrhizal fungus (AMF) *Funneliformis mosseae*. Bars within panels that do not share the same letter are significantly different from each other (post-hoc tests using LS means, P < 0.05).


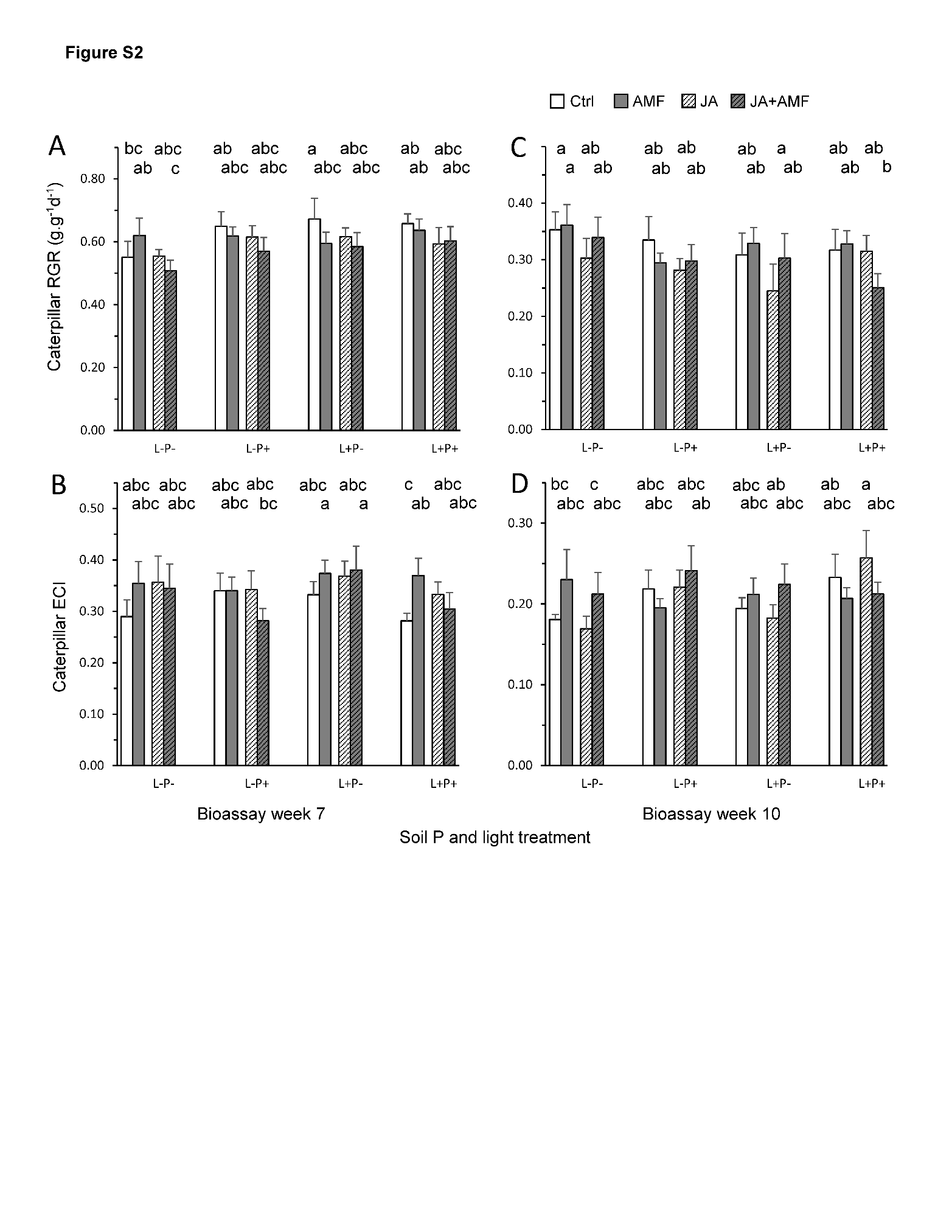


**Supplementary Figure S2.** Growth parameters of *Mamestra brassicae* caterpillars feeding on *Plantago lanceolata* leaves during two bioassays, performed when plants were 7 weeks (left panels), and 10 weeks old (right panels). (**A,C**) RGR (Relative Growth Rate; amount of caterpillar weight produced per unit of caterpillar weight per day); (**B,D**) ECI (Efficiency of Conversion of Ingested food; amount of caterpillar weight produced per unit of leaf weight eaten). Each panel displays results for plants grown under four combinations of light intensity (L-: low light; L+: high light) and soil phosphorus treatments (P-: low soil P; P+: high soil P). In addition, plants had either be challenged by leaf application of jasmonic acid (JA) 24 hrs. prior to the bioassay (hatched bars) or not (non-hatched bars) and plants had either been inoculated with the arbuscular mycorrhizal fungus (AMF) *Funneliformis mosseae* (bars with grey background) or not (white background). Bars within panels that do not share the same letter are significantly different from each other (post-hoc tests using LS means, P < 0.05).

**
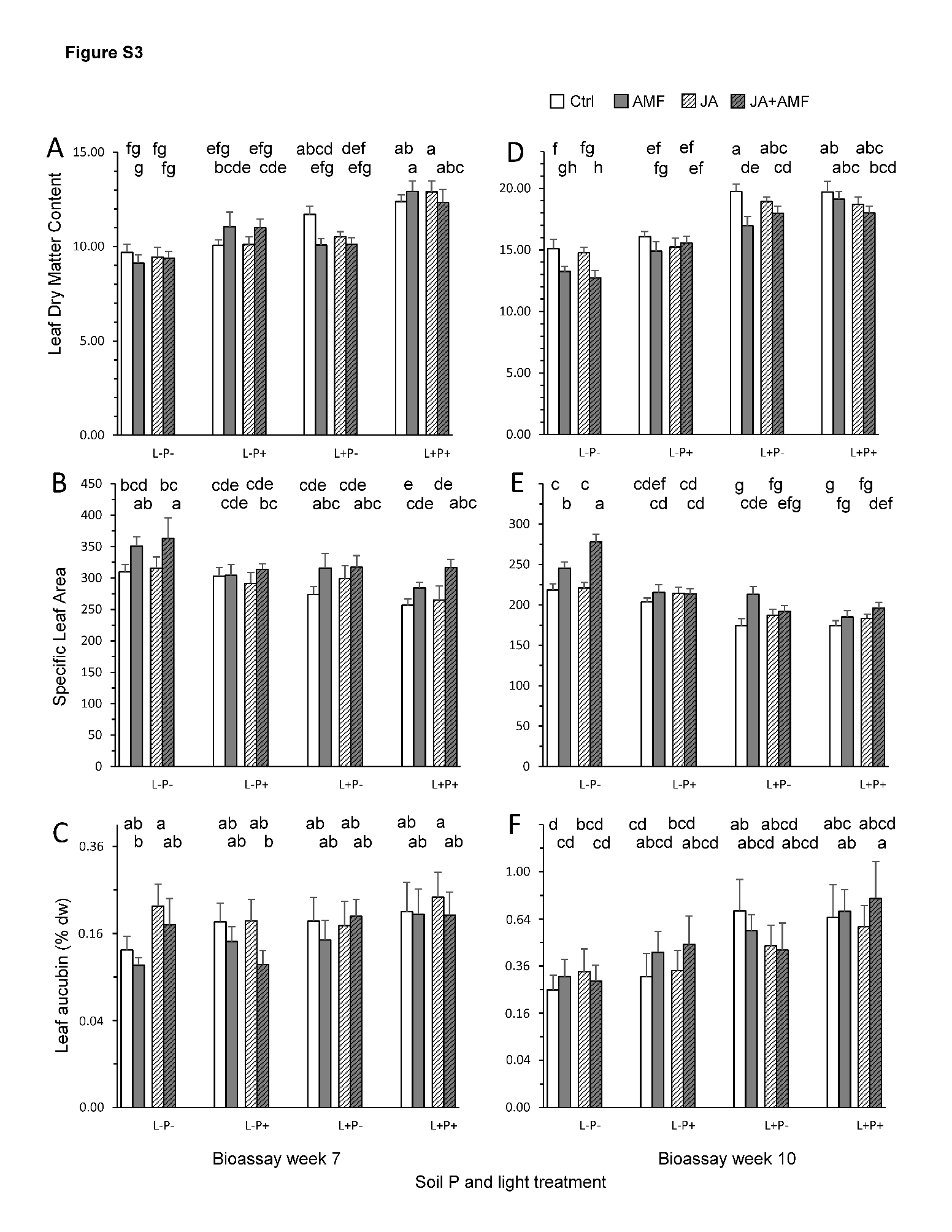
**

**Supplementary Figure S3**. Leaf characteristics of *Plantago lanceolata* plants at the time of two bioassays, when plants were 7 (left panels) and 10 weeks (right panels) old. **(A,D)** LDMC (Leaf Dry Matter Content; leaf dry weight divided by leaf fresh weight); **(B,E)** SLA (Specific Leaf Area; leaf area per unit leaf dry weight); **(C,F)** Leaf concentration of the defense chemical aucubin. Each panel displays results for plants grown under four combinations of light intensity (L-: low light; L+: high light) and soil phosphorus treatments (P-: low soil P; P+: high soil P). In addition, plants had either be challenged by leaf application of jasmonic acid (JA) 24 hrs. prior to the bioassay (hatched bars) or not (non-hatched bars) and plants had either been inoculated with the arbuscular mycorrhizal fungus (AMF) *Funneliformis mosseae* (bars with grey background) or not (white background). Bars within panels that do not share the same letter are significantly different from each other (post-hoc tests using LS means, P < 0.05).
